# Supplementary figures and images for: Object color knowledge representation occurs in the macaque brain despite the absence of a developed language system
Source: PLoS Biol. 2024 Oct 28;22(10):e3002863. doi: 10.1371/journal.pbio.3002863 (PMC11542842; doi:10.1371/journal.pbio.3002863)

(A)

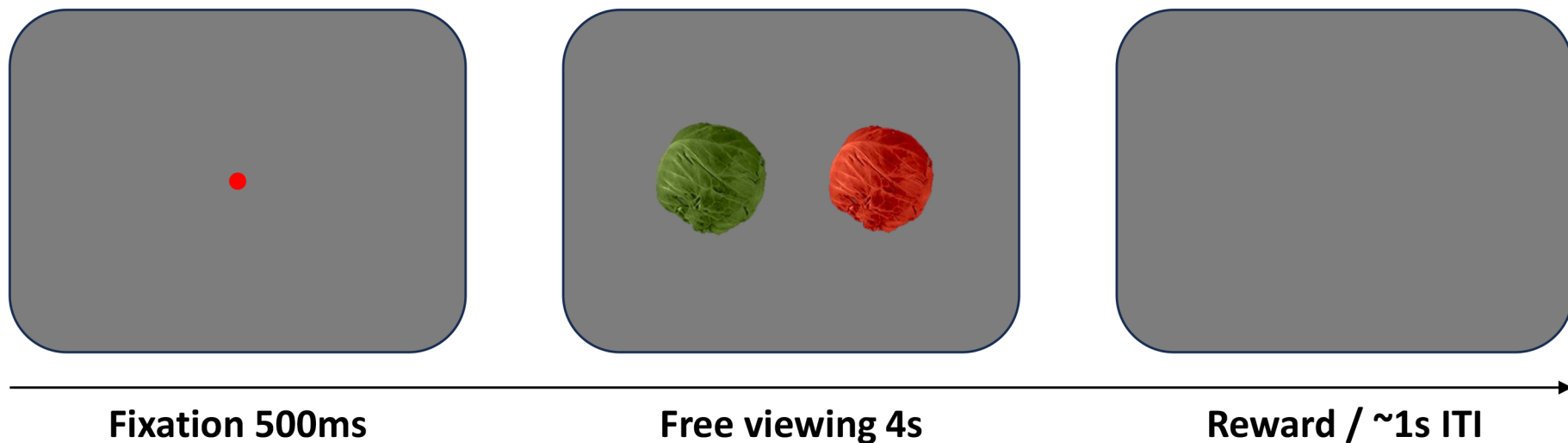

(B)

### Average proportion of fixation time

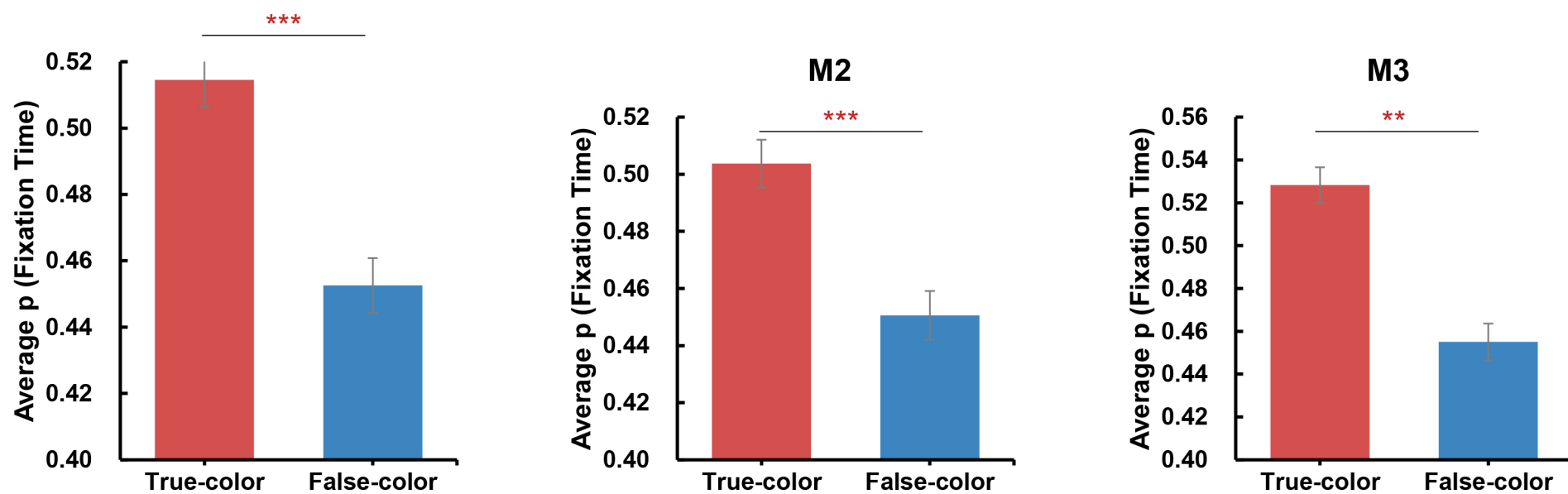

(C)

### Average proportion of first fixation

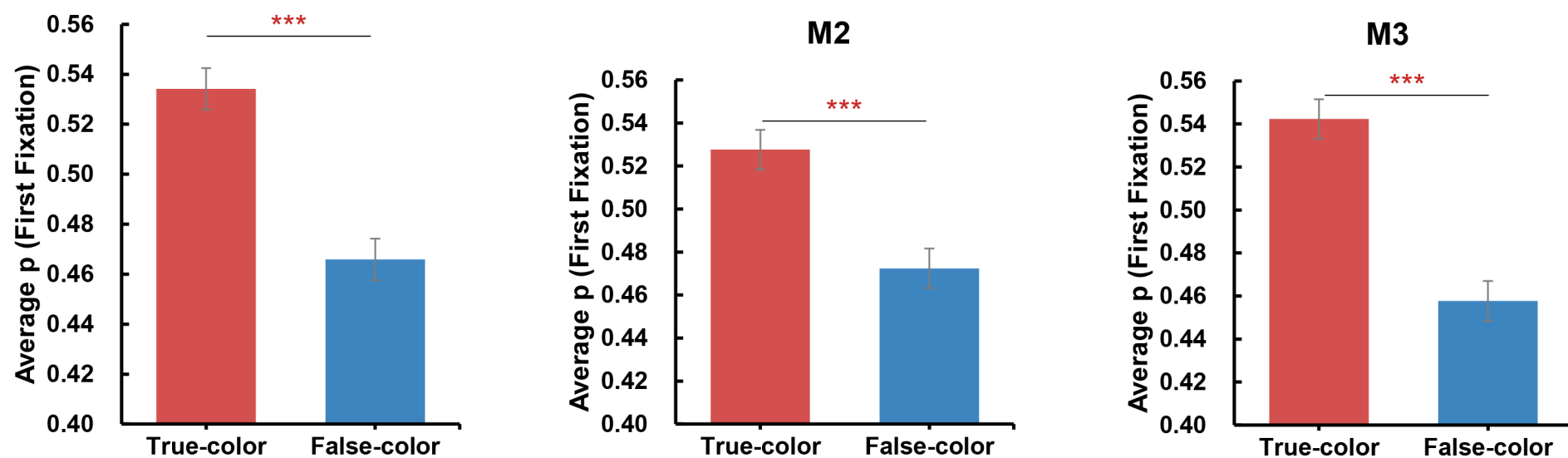

Supplement: S1 Fig — (A) The trial procedure: fixation, free viewing, and reward after successful trial completion. (B) From left to right, the proportion of fixation time averaged across 2 monkeys and for each monkey. (C) From left to right, the proportion of first fixation averaged across 2 monkeys and for each monkey. Bars display mean values +/− SEM. Red asterisks indicate a significant difference between responses evoked by true- and false-colored stimuli in B and C; **p < 0.01, ***p < 0.001. The data underlying this figure are available in S1 Data. (PDF) [file pbio.3002863.s001.pdf]

(A)

**Chromatic grating > Achromatic grating**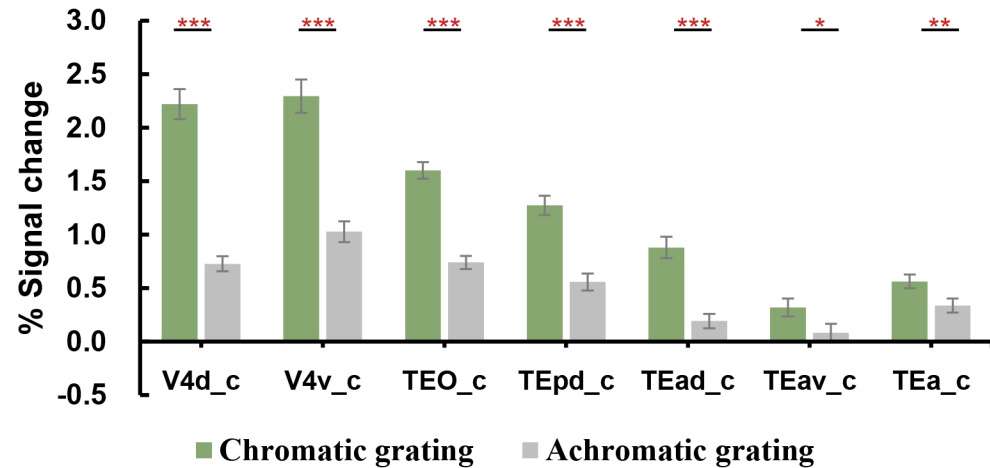

(B)

**Real color decoding**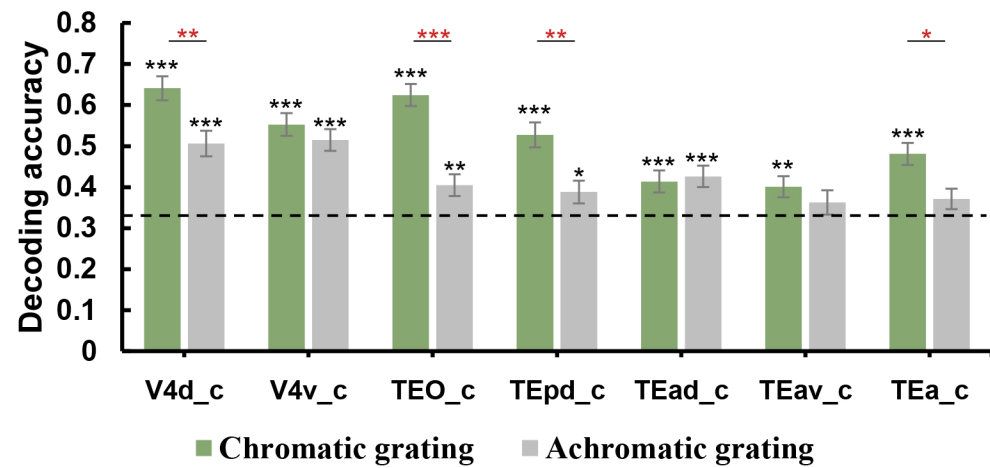

Supplement: S2 Fig — (A) Averaged fMRI responses elicited by chromatic and achromatic gratings, which were not used to define color patches, in color patches across all 3 subjects. (B) Chromatic and achromatic decoding accuracy when training the classifier to distinguish among the 3 chromatic/achromatic gratings in N-1 runs and testing on the left-out run. Black asterisks indicate a significant difference from the chance level (0.333 in B, indicated by the dash lines), and red ones indicate a significant difference between chromatic and achromatic gratings; *q < 0.05, **q < 0.01, ***q < 0.001. The data underlying this figure are available in S1 Data. (PDF) [file pbio.3002863.s002.pdf]

(A)

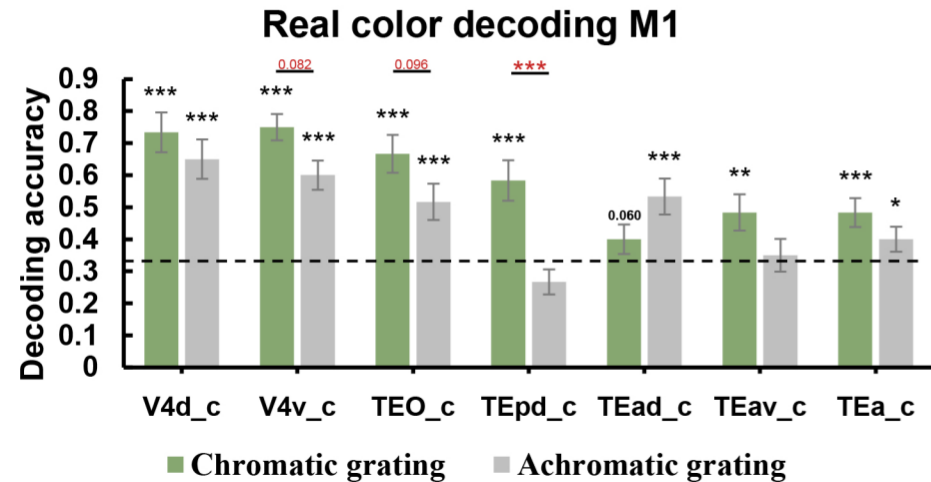

(B)

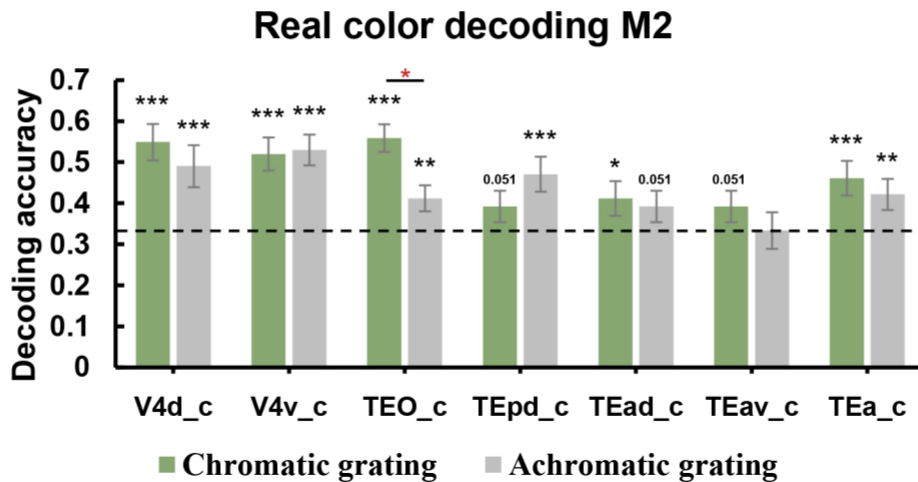

(C)

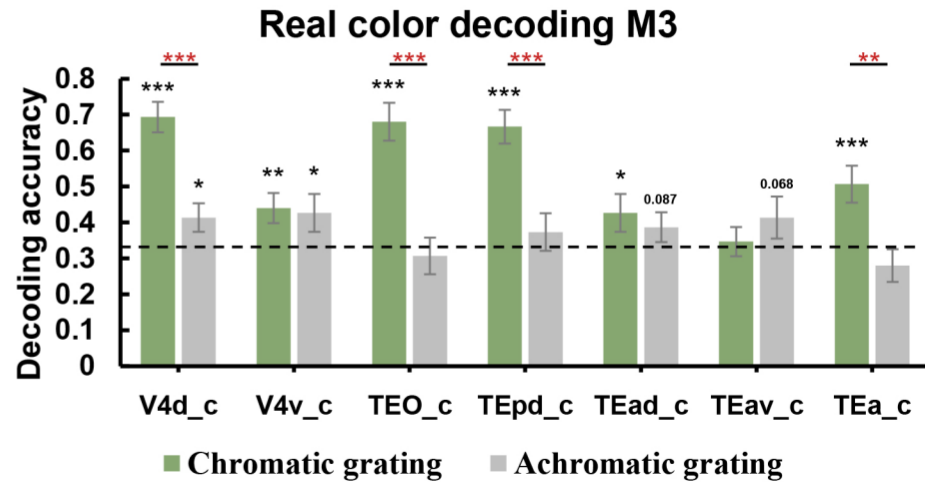

Supplement: S3 Fig — Bars display mean values +/− SEM. Black asterisks indicate a significant difference from the chance level (0.333, indicated by the dash lines), and red ones indicate a significant difference between chromatic and achromatic gratings; *p < 0.05, **p < 0.01, ***p < 0.001. The numbers above the bars indicate p-values that are marginally significant (p < 0.1). The data underlying this figure are available in S1 Data. (PDF) [file pbio.3002863.s003.pdf]

(A)

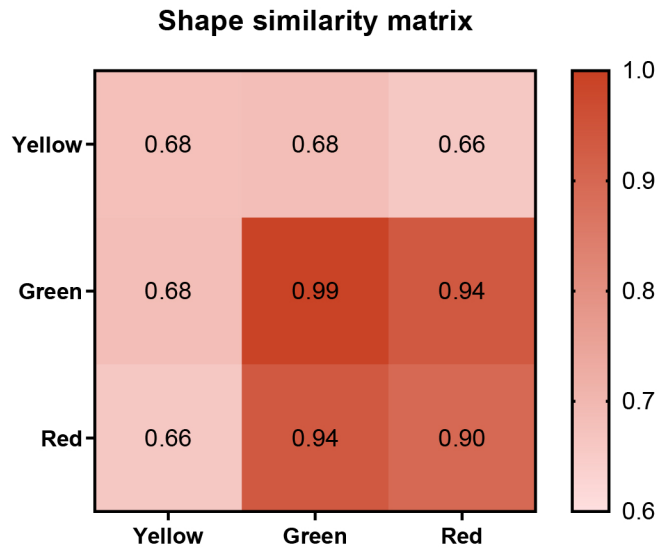

(B)

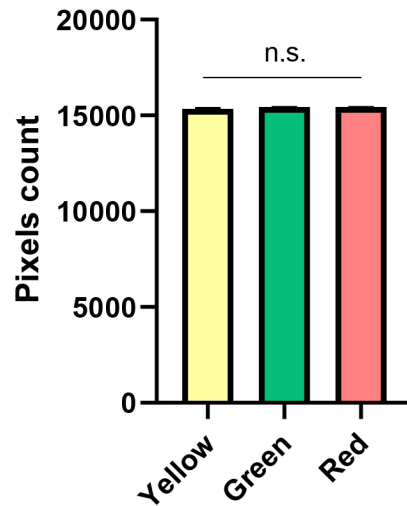

(C)

Luminance Histograms

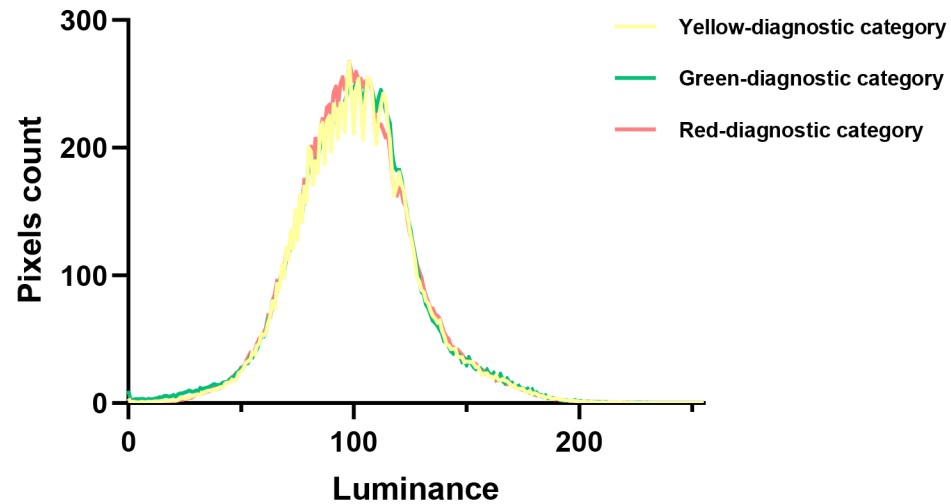

Supplement: S6 Fig — (A) The averaged shape similarity matrix of grayscale images from different color categories. (B) Pixel counts of foreground of grayscale images from different color categories. Bars display mean values +/− SEM. (C) Luminance histograms of foreground of grayscale images from different color categories. The data underlying this figure are available in S1 Data. (PDF) [file pbio.3002863.s006.pdf]

(A)

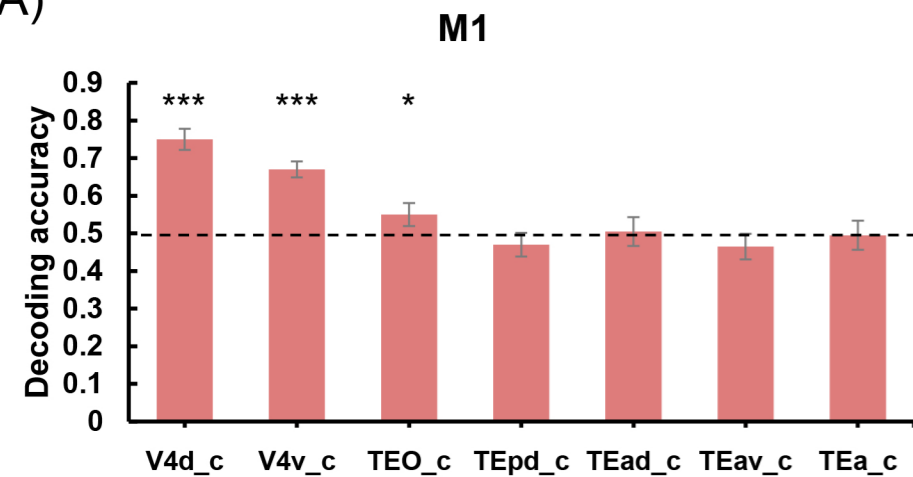

(B)

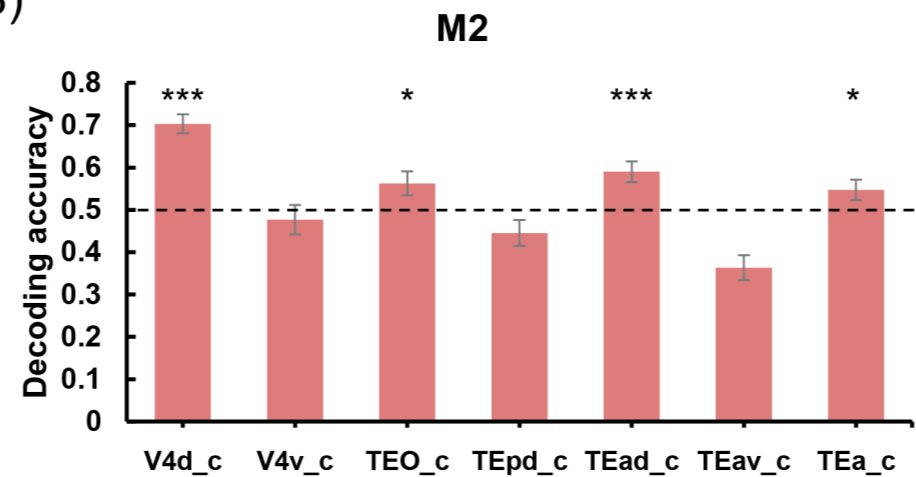

(C)

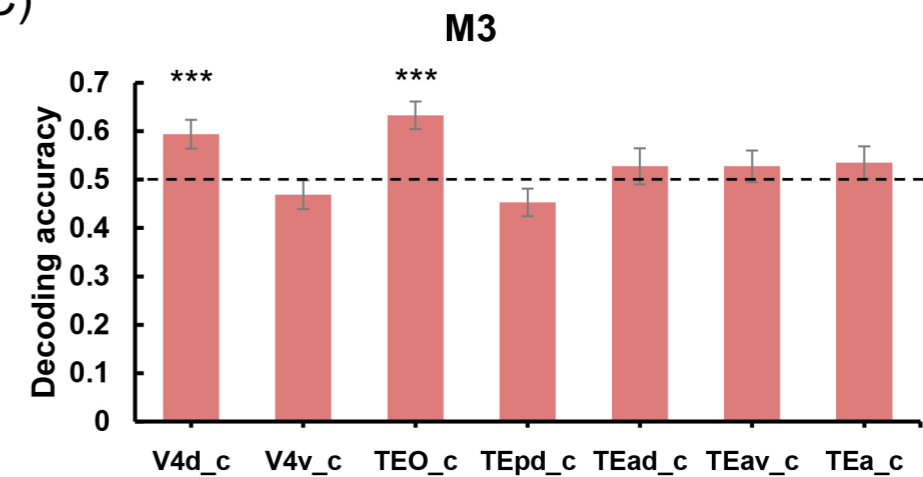

(D)

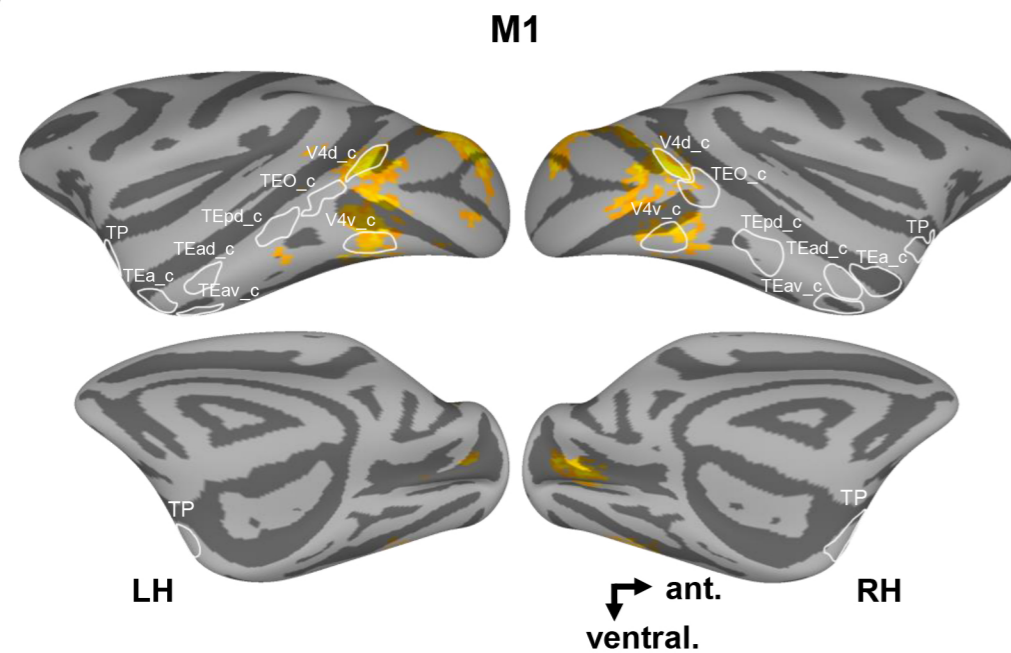

(E)

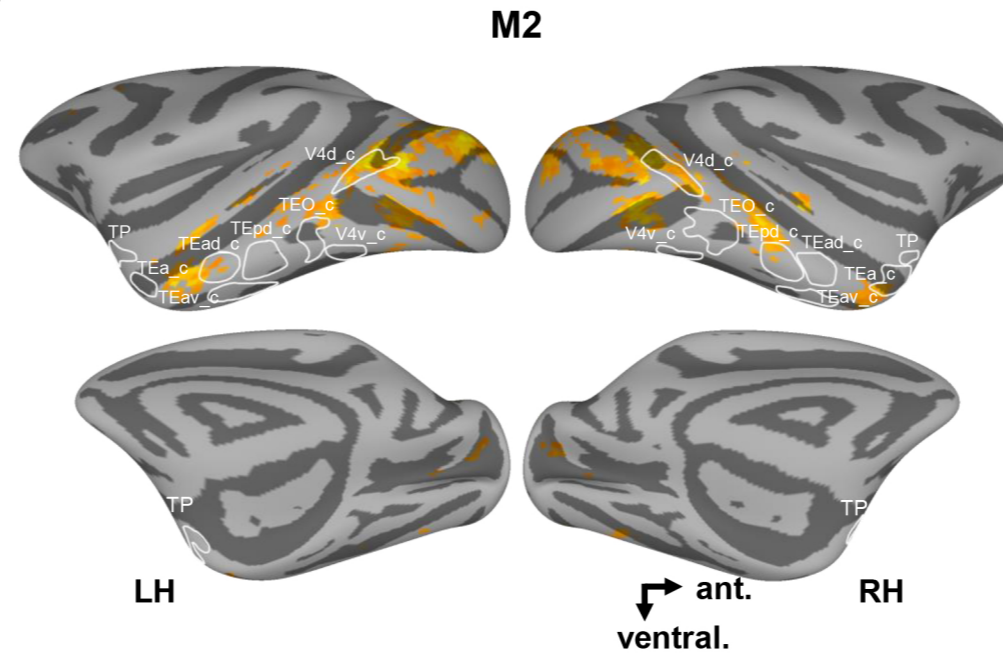

(F)

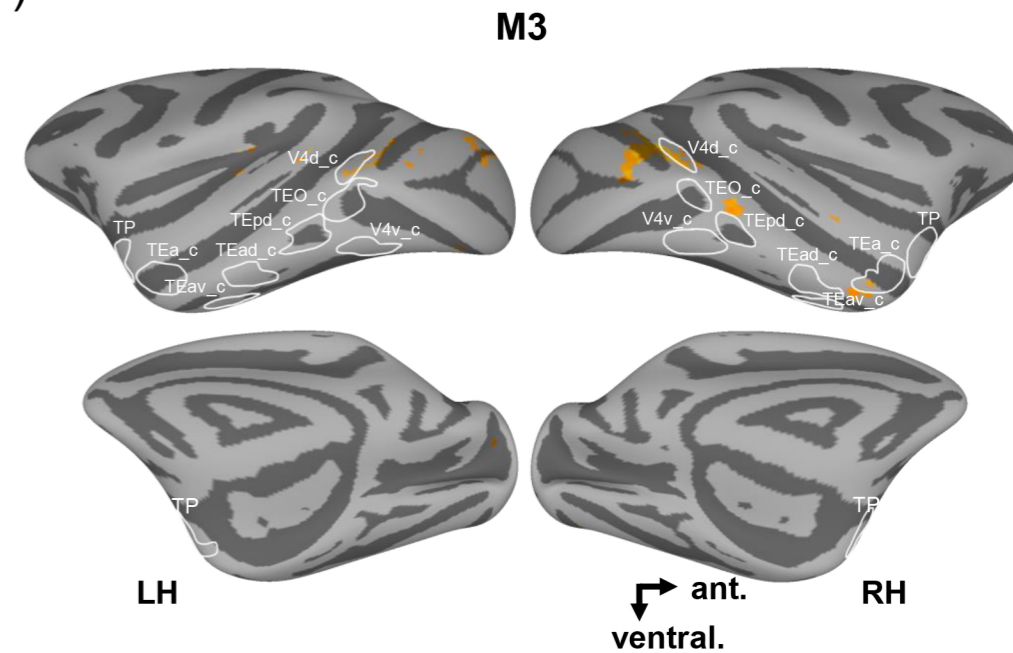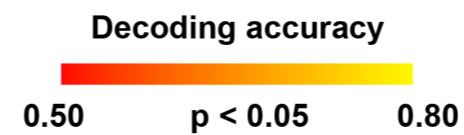

Supplement: S7 Fig — (A–C) Classification of grayscale objects with red and green memory colors in each monkey. Bars display mean values +/− SEM. Black asterisks indicate a significant difference from the chance level (0.5, indicated by the dashed lines); *p < 0.05, **p < 0.01, ***p < 0.001. (D–F) The results of whole-brain searchlight analyses for decoding memory color in each subject shown on the template inflated surface. White solid lines indicate color patches and TP defined for each subject. The data underlying this figure are available in S1 Data. (PDF) [file pbio.3002863.s007.pdf]

(A)

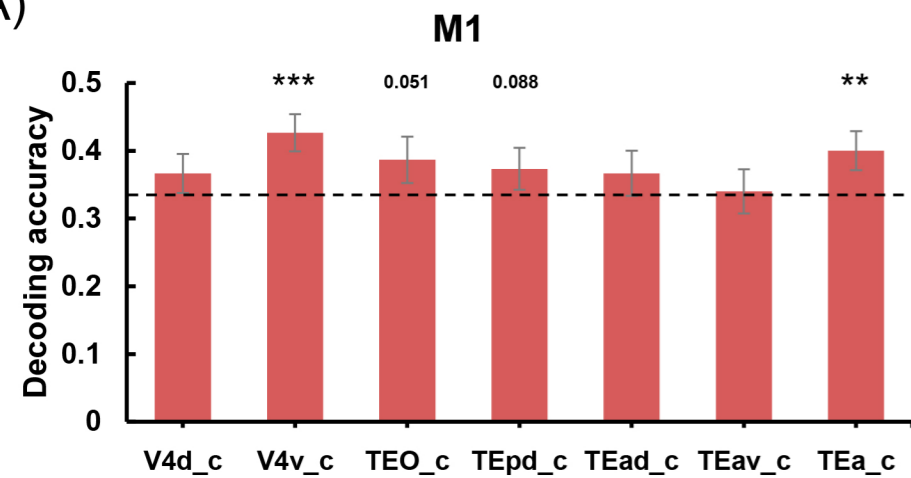

(B)

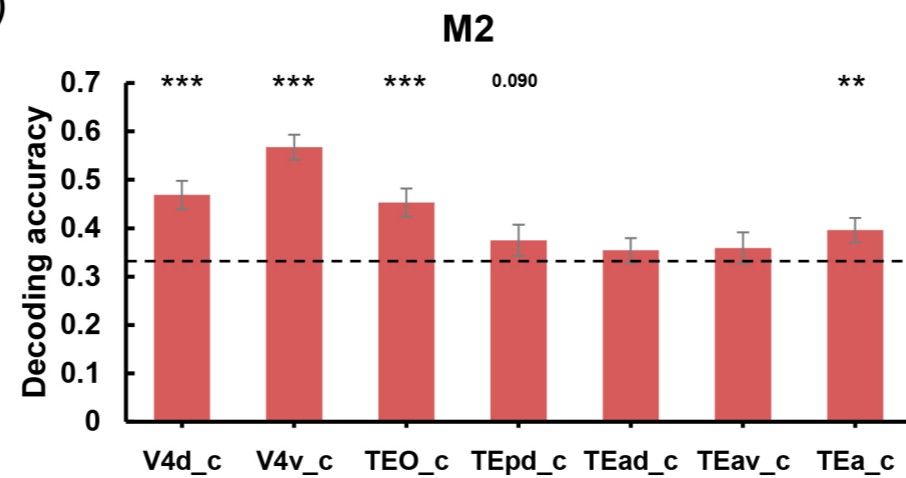

(C)

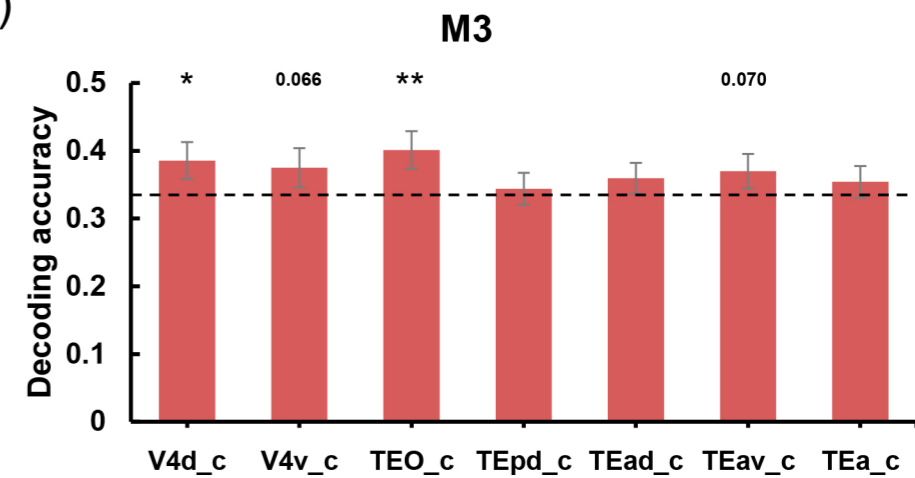

(D)

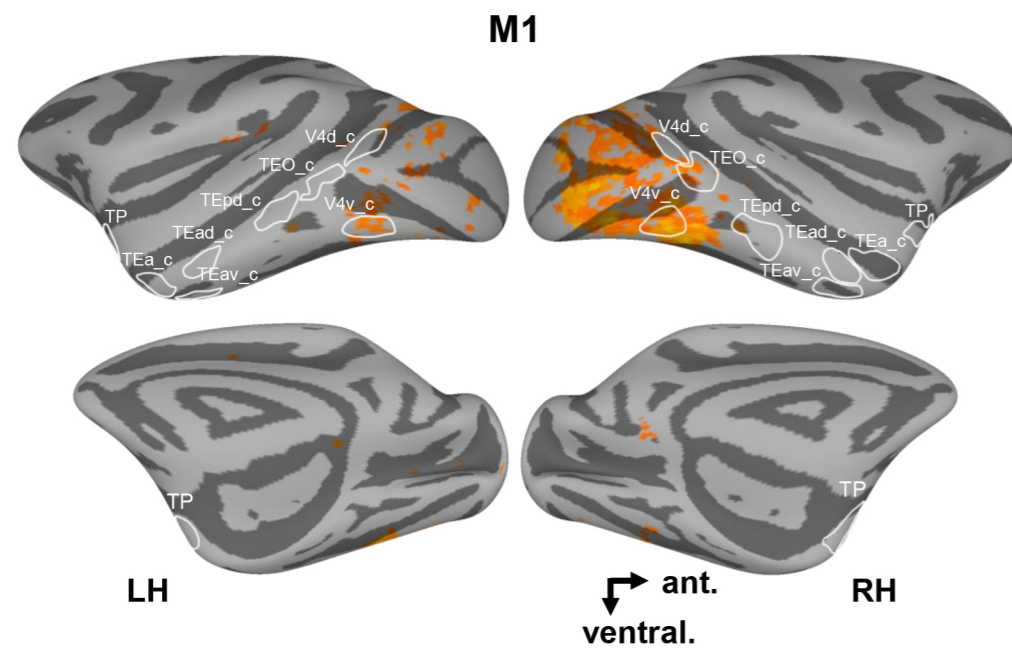

(E)

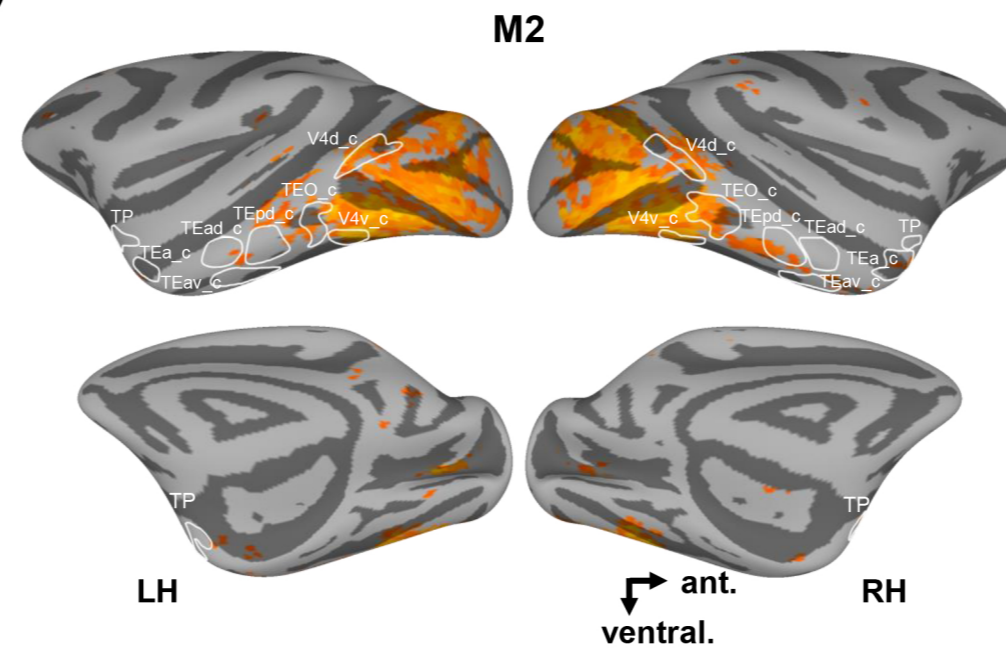

(F)

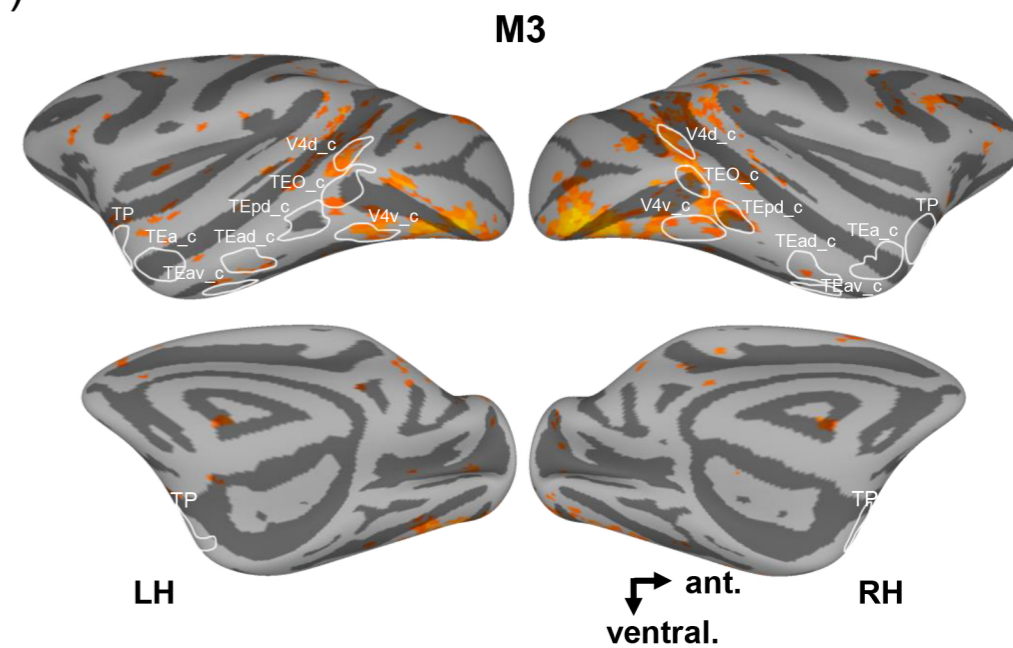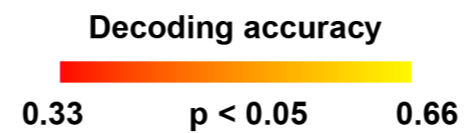

Supplement: S8 Fig — (A–C) Memory color decoding accuracy in color patches in each monkey. Bars display mean values +/− SEM. Black asterisks indicate a significant difference from the chance level (0.333, indicated by the dashed lines); *p < 0.05, **p < 0.01, ***p < 0.001. The numbers above the bars indicate p-values that are marginally significant (p < 0.1). (D–F) The results of whole-brain searchlight analyses for decoding memory color in each subject shown on the template inflated surface. White solid lines indicate color patches and TP defined for each subject. The data underlying this figure are available in S1 Data. (PDF) [file pbio.3002863.s008.pdf]

True-False color responses

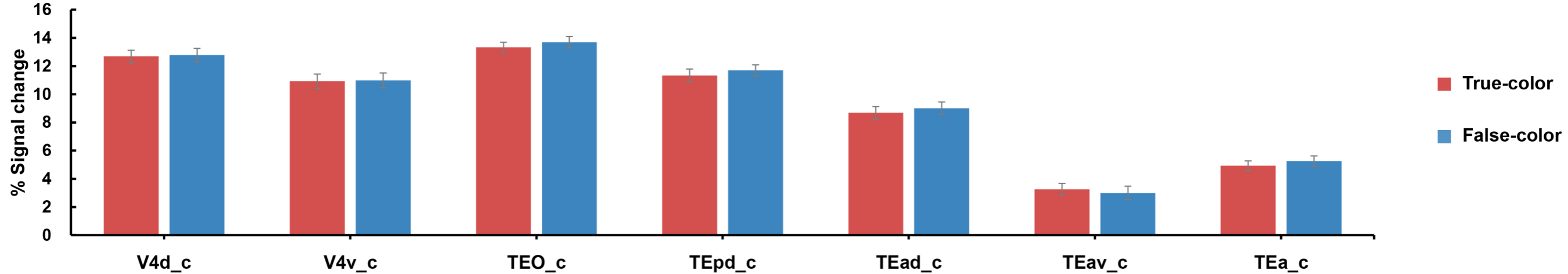

Supplement: S10 Fig — No significant differences were found between responses evoked by true- and false-color objects. Bars display mean values +/− SEM. The data underlying this figure are available in S1 Data. (PDF) [file pbio.3002863.s010.pdf]

(A)

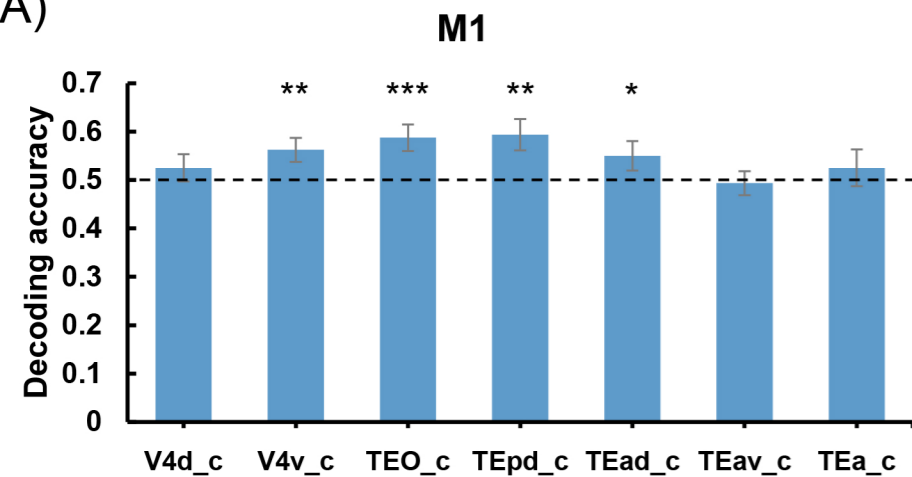

(B)

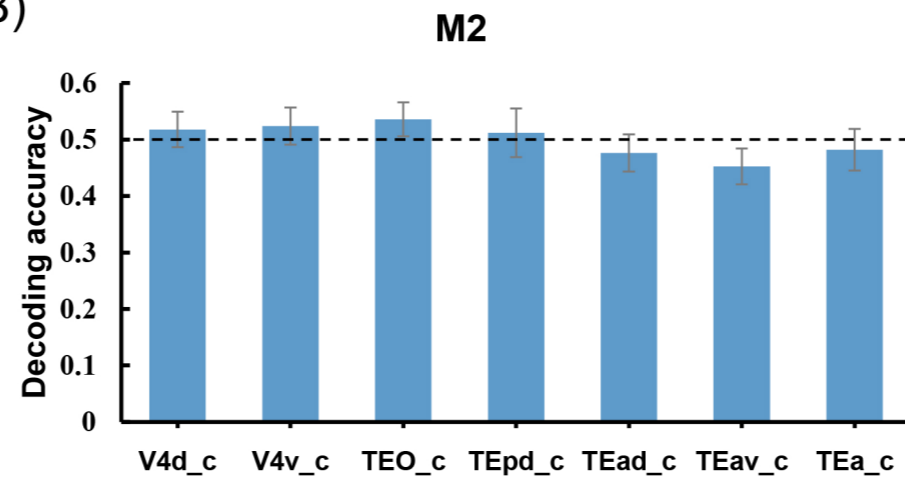

(C)

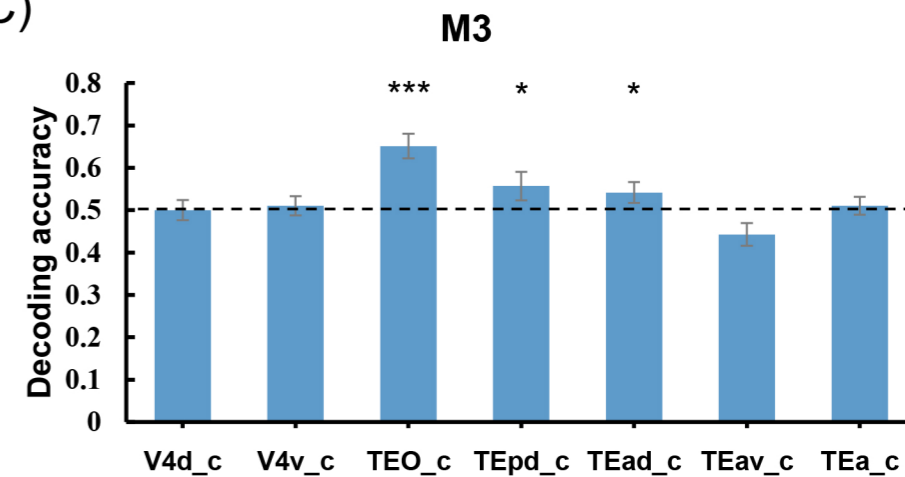

(D)

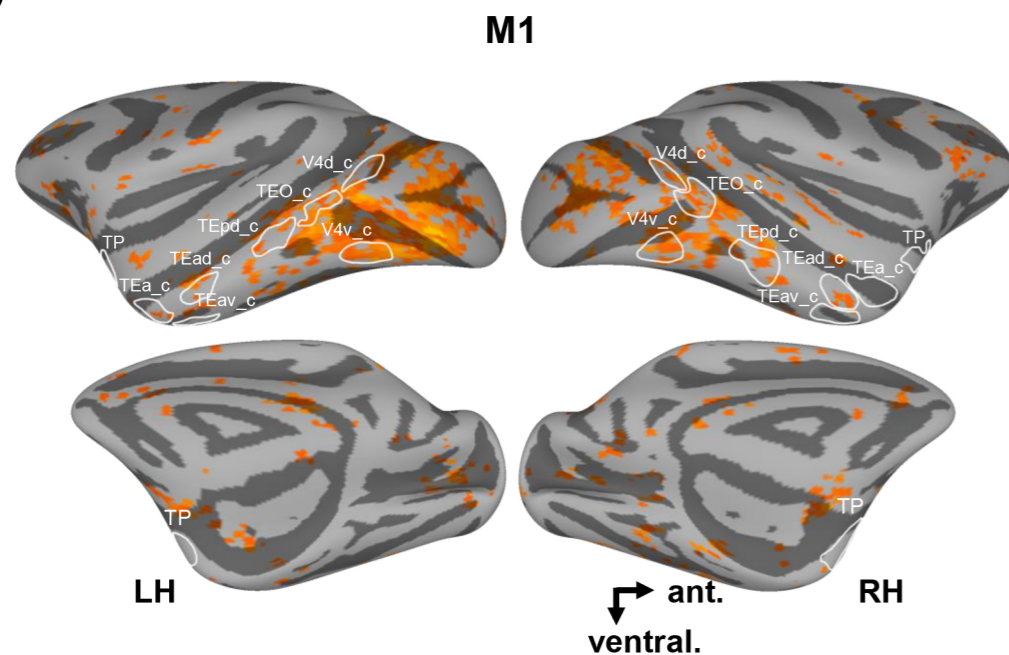

(E)

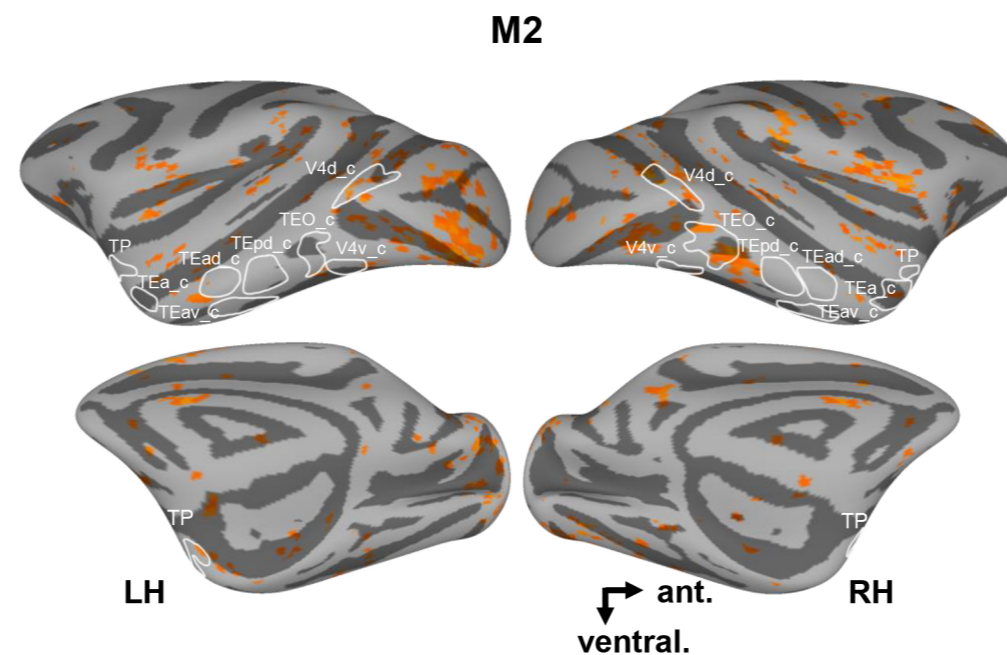

(F)

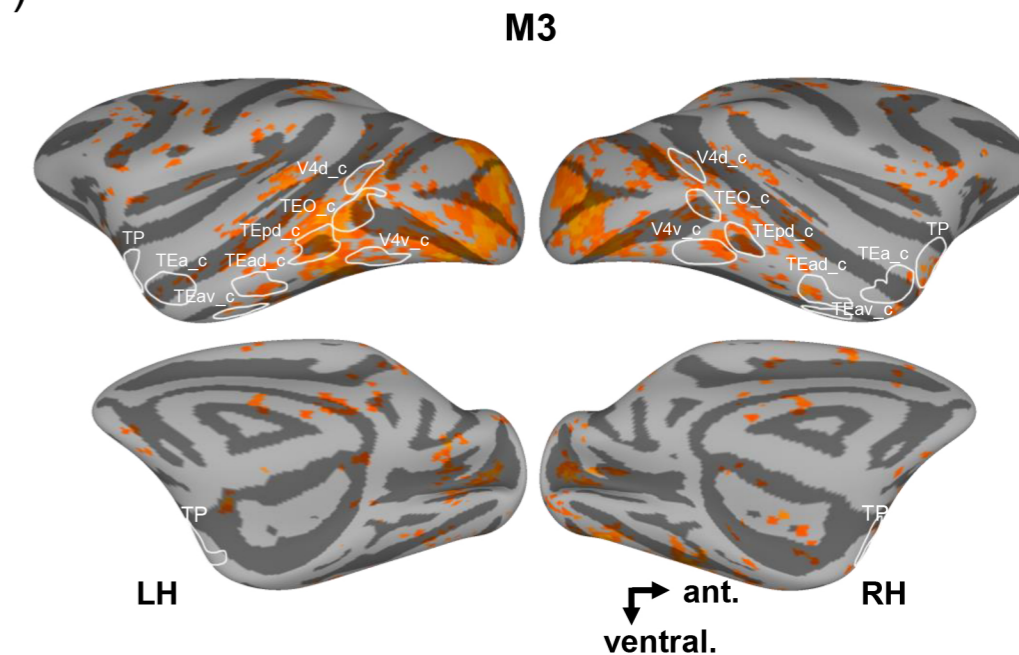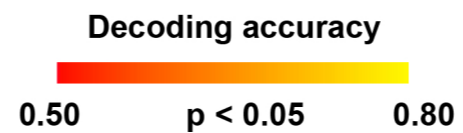

Supplement: S11 Fig — (A–C) True-False color decoding accuracy in color patches in each monkey. Bars display mean values +/− SEM. Black asterisks indicate a significant difference from the chance level (0.5, indicated by the dashed lines); *p < 0.05, **p < 0.01, ***p < 0.001. (D–F) The results of whole-brain searchlight analyses for decoding true-false color for each subject shown on the template inflated surface. White solid lines indicate color patches and TP defined for each subject. The data underlying this figure are available in S1 Data. (PDF) [file pbio.3002863.s011.pdf]

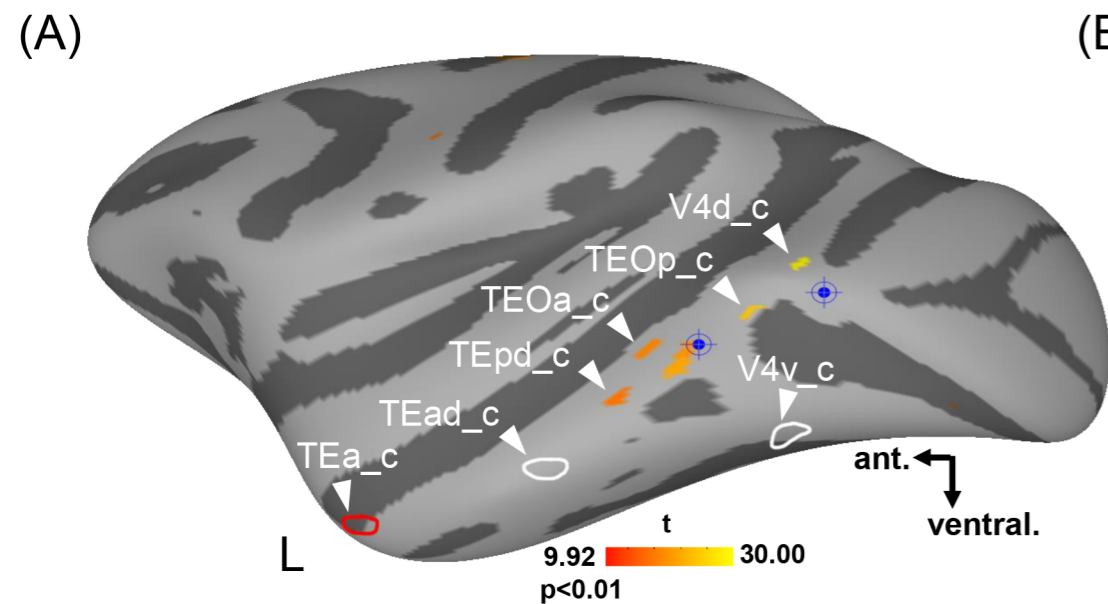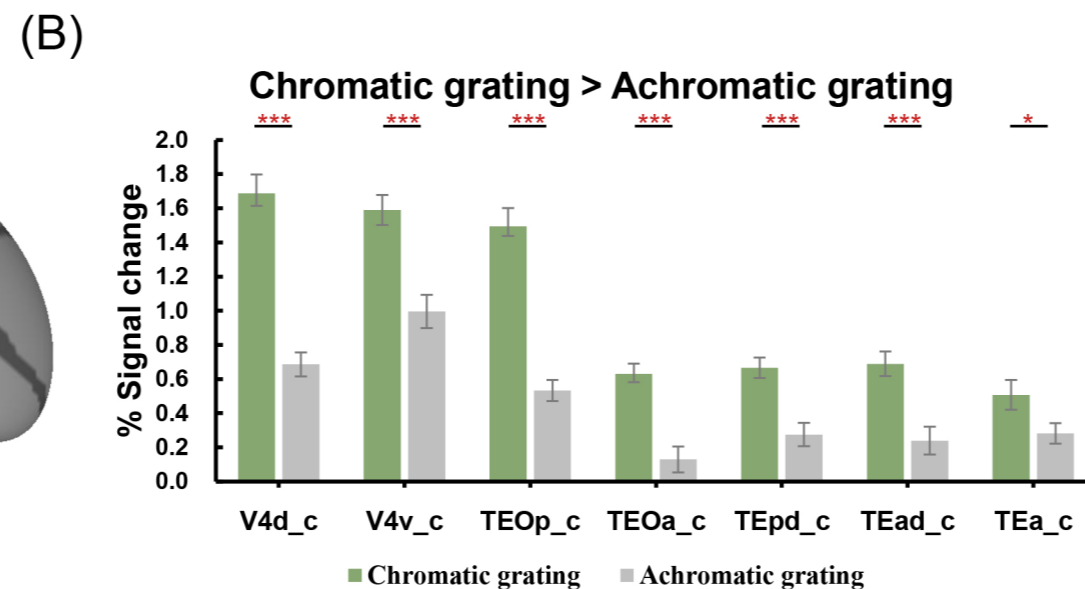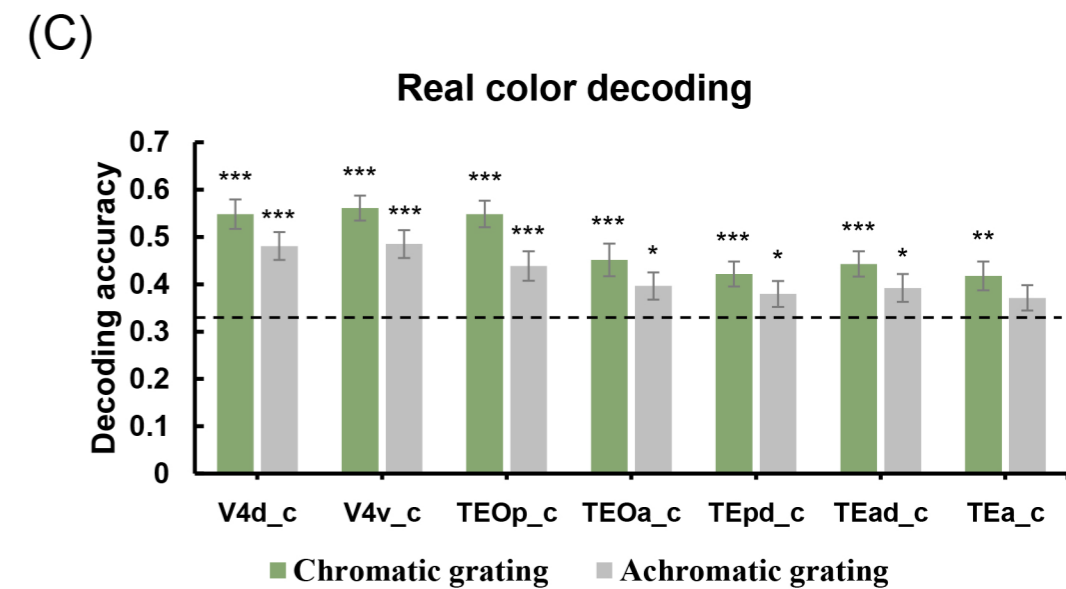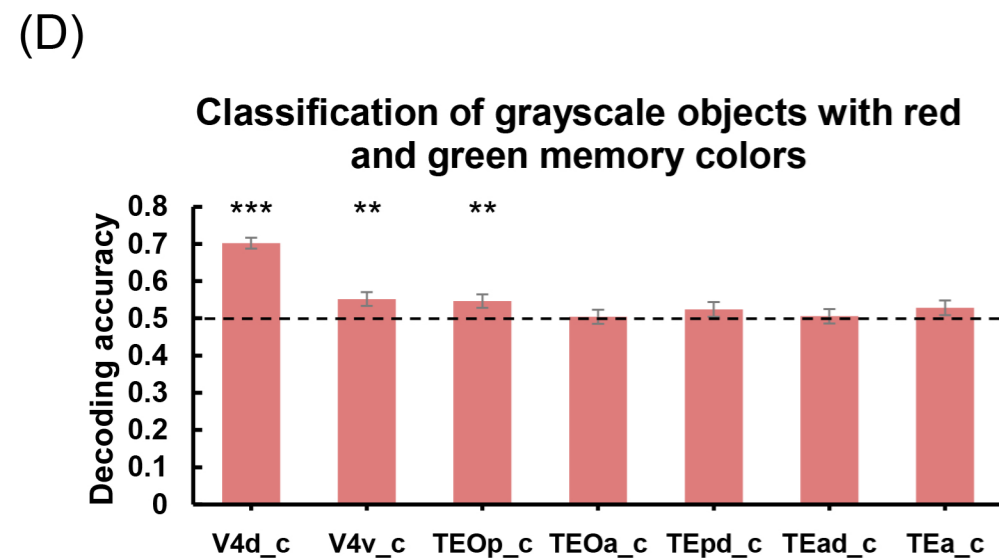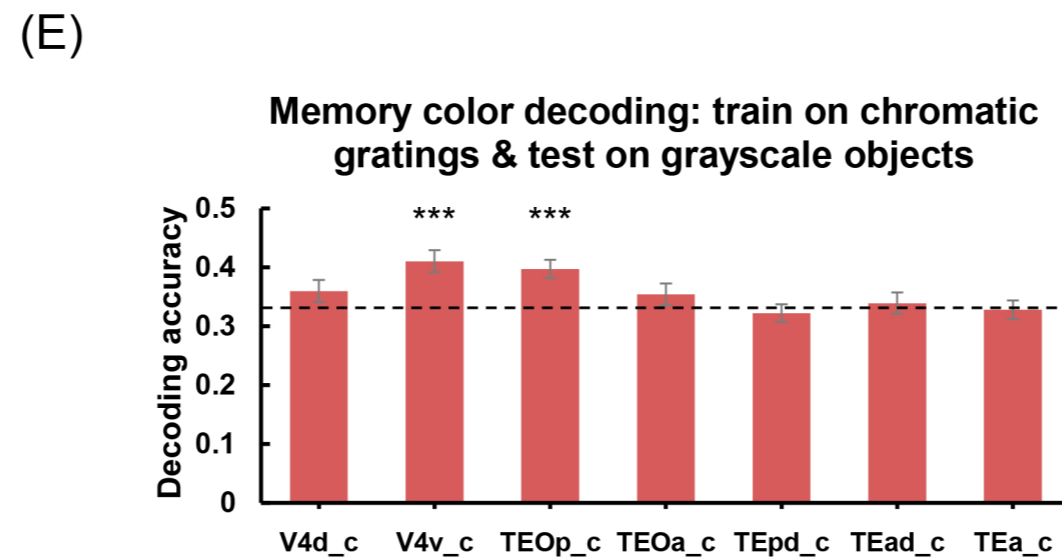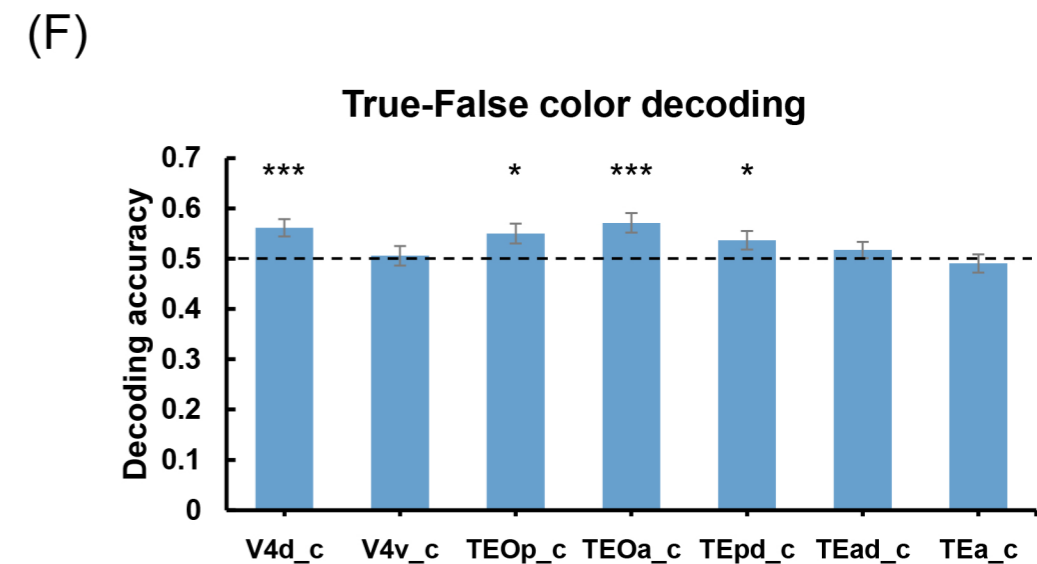

Supplement: S12 Fig — (A) Color biased clusters defined by the group analysis based on the 6 hemispheres from the 3 subjects (p < 0.01, uncorrected) from Exp 1 are shown on the lateral view of the template inflated surface of the left hemisphere. Red solid lines indicate that clusters could be defined at p < 0.05 (uncorrected), while white solid lines indicate clusters that could be defined when adjusting the contrasted achromatic grating to the next lower level (i.e., from 50% to 25% in M1 and M2, p < 0.01, uncorrected) to account for the relatively weak color bias in the right hemisphere. Blue bullseye marks the approximate location of the fovea based on the data set shared by Janssens and colleagues [94], which was downloaded from https://gbiomed.kuleuven.be/english/research/50000666/50000669/50488669/neuroserv/publications/JNEUROSCI. The permission to use dataset from Janssens and colleagues [94](https://www.jneurosci.org/content/34/31/10156) has been granted by the corresponding author, Wim Vanduffel. (B) Averaged fMRI responses elicited by chromatic and achromatic gratings, which were not used to define color patches, in color patches defined by the group analysis across 3 monkeys. Red asterisks indicate a significant difference between chromatic and achromatic gratings; *q < 0.05, ***q < 0.001. (C) Chromatic and achromatic decoding accuracy when training the classifier to distinguish among the 3 chromatic/achromatic gratings in N-1 runs and testing on the left-out run in Exp 1. (D) Results of classification of grayscale objects with red and green memory colors: training the classifier to distinguish half set of the red and green color-diagnostic grayscale objects and testing on the other half in Exp 2. (E) Results of memory color decoding based on chromatic gratings training: training the classifier to distinguish among 3 chromatic gratings in Exp 1 and then testing on 3 categories of grayscale objects in Exp 2. (F) Results of true-false color decoding: training on true- and false [file pbio.3002863.s012.pdf]

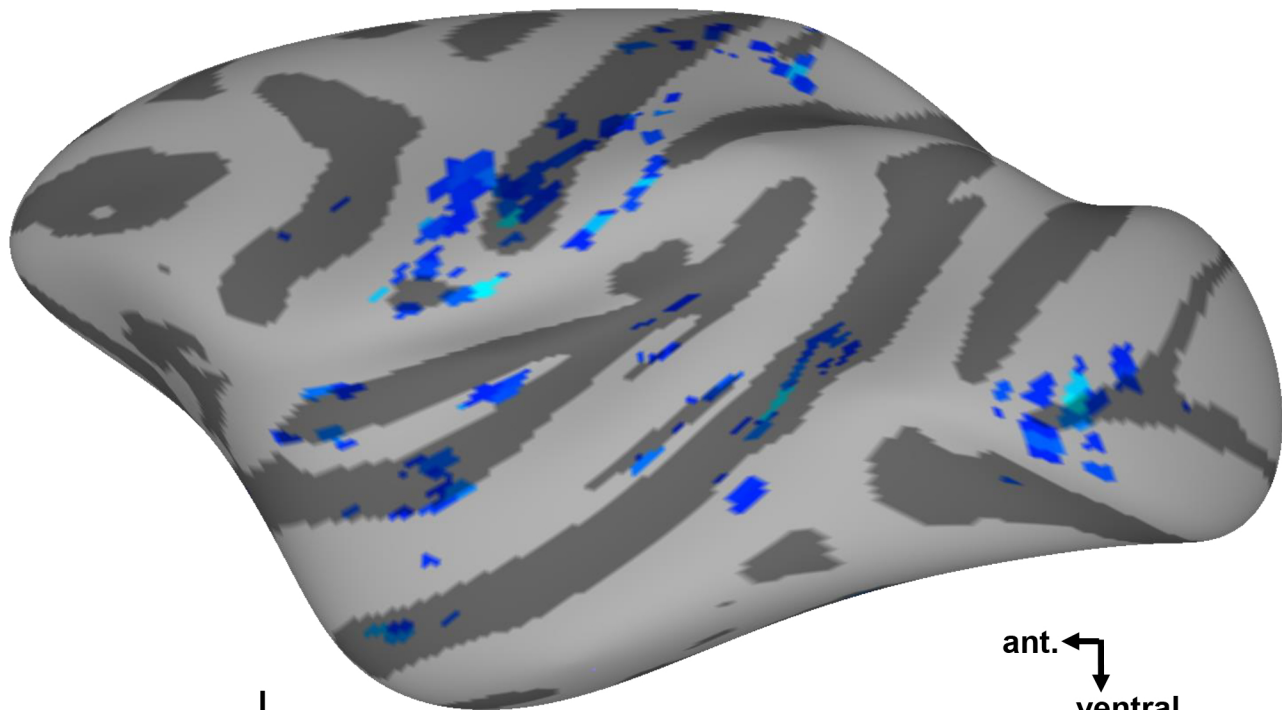

L

ant. 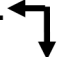  
ventral.

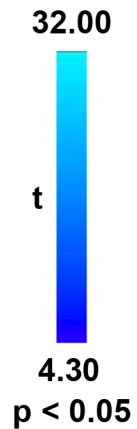

Supplement: S14 Fig — Results of the whole-brain analysis at the group level are shown on the lateral view of the template inflated surface. (PDF) [file pbio.3002863.s014.pdf]

(A)

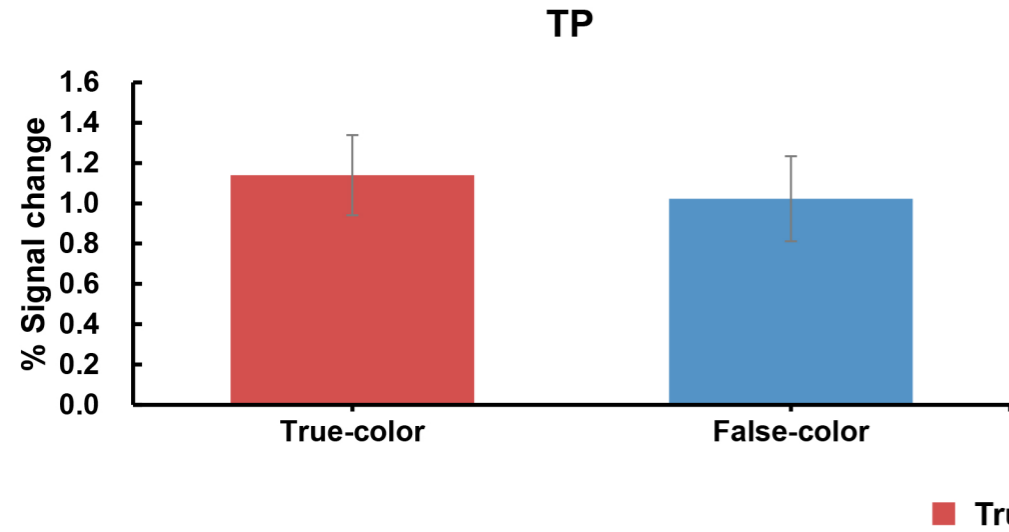

(B)

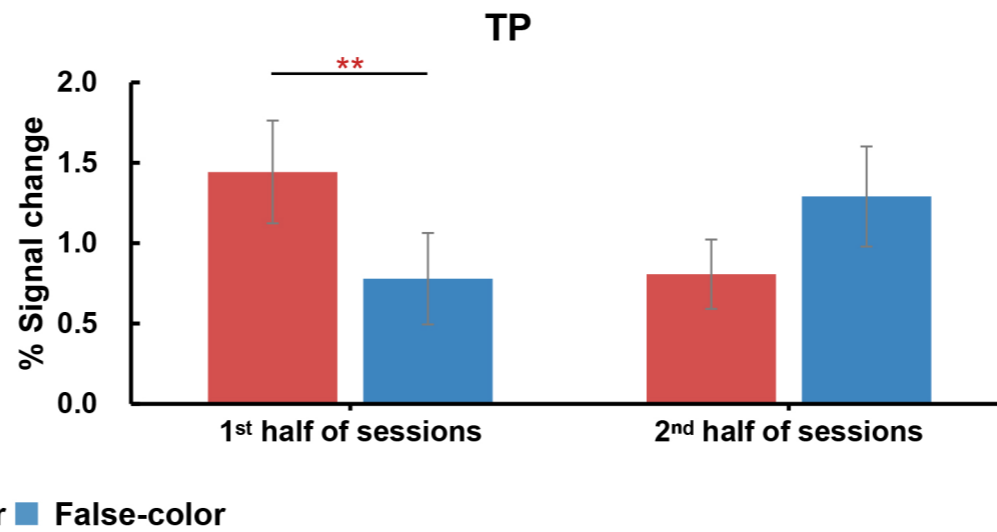

(C)

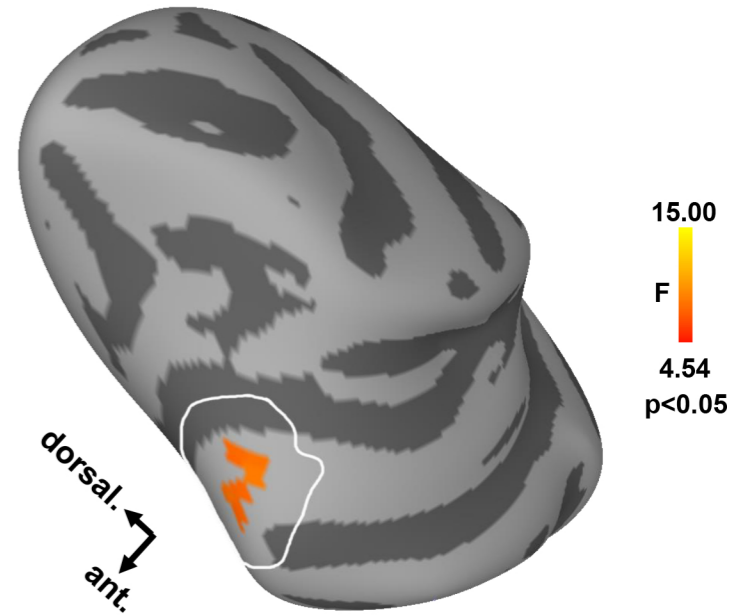

Supplement: S16 Fig — (A) Averaged fMRI responses to true- and false-colored objects in TP across all sessions and subjects. To avoid the possible circularity, we used a one-half set of the true- and false-colored objects to define the true-false ROI in TP and then examined the true-false differentiation effect on the other half set of the stimuli. Due to this strict approach, differences between true- and false-colored objects when combining all sessions were not visible [F(1,128) = 0.274, q = 0.823, η2 = 0.002; two-tailed]. (B) Averaged fMRI responses to true- and false-colored objects in TP across all 3 subjects from the first and second halves of sessions. We did observe an interesting learning effect: a significant interaction effect between Period and True-False across 3 monkeys [F(1,126) = 11.006, q = 0.002, η2 = 0.080; two-tailed]. To further investigate this interaction effect, we conducted post hoc analyses. In the first half of the sessions, the ROI analysis revealed that TP responded significantly more strongly to true-colored objects across 3 monkeys [t(126) = 3.420, q = 0.002, Cohen’s d = 0.609; two-tailed]. The differences in averaged responses evoked by true- and false-colored objects across all 3 monkeys observed in the first half of the sessions vanished in the second half. Bars display mean values +/− SEM. Red asterisks indicate a significant difference between responses evoked by true- and false-colored stimuli; **q < 0.01. (C) Regions exhibiting the interaction effect between Period (first half versus second half of sessions) and True-False of the whole-brain analysis at the group level are shown on the lateral view of the template inflated surface. White solid lines indicate the location of TP from the D99 atlas [76,80]. The data underlying this figure are available in S1 Data. (PDF) [file pbio.3002863.s016.pdf]

(A)

M1

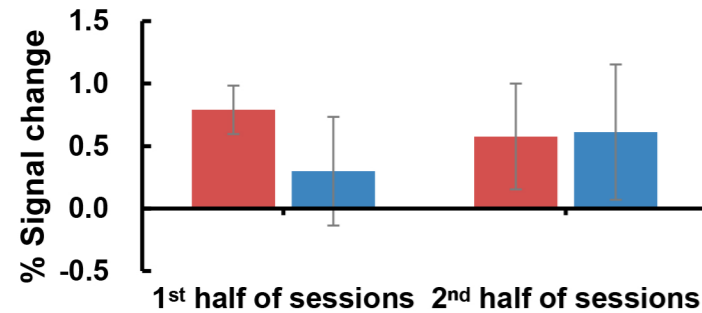

(B)

M2

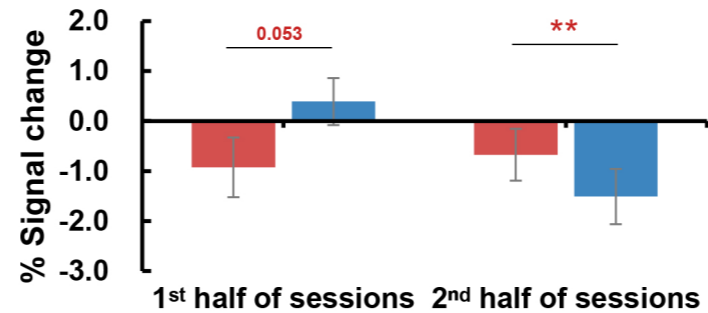

True-color False-color

(C)

M3

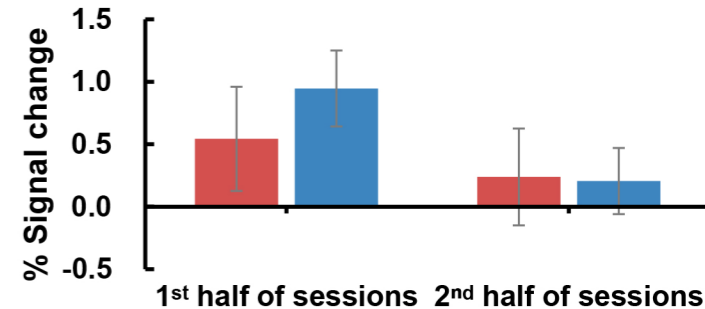

Supplement: S17 Fig — (A–C) FMRI responses to true- and false-colored objects in PR from the first and second halves of sessions for each subject, respectively. Bars display mean values +/− SEM. Red asterisks indicate a significant difference between responses evoked by true- and false-colored stimuli; **p < 0.01. The numbers above the bars indicate p-values that are marginally significant (p < 0.1). The data underlying this figure are available in S1 Data. (PDF) [file pbio.3002863.s017.pdf]

(A)

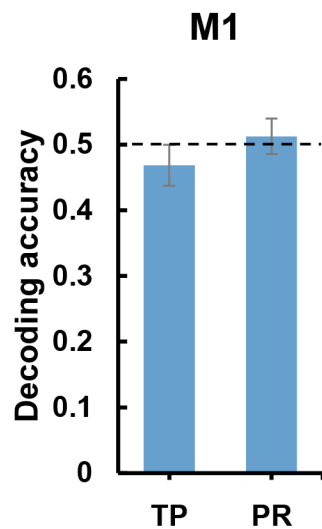

(B)

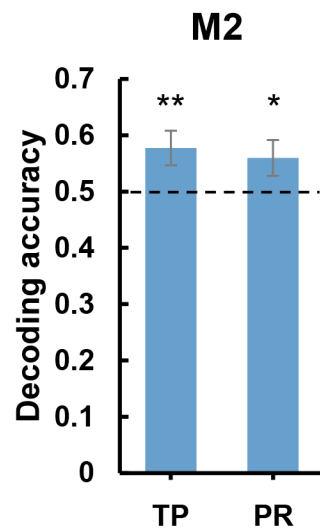

(C)

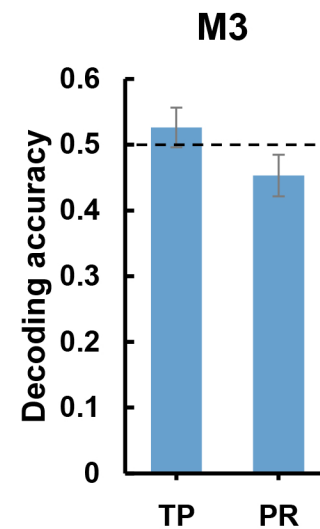

(D)

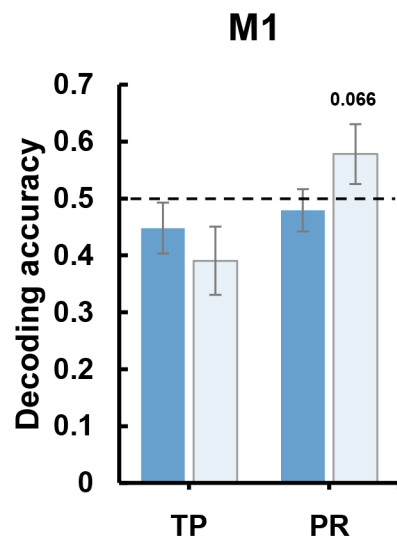

(E)

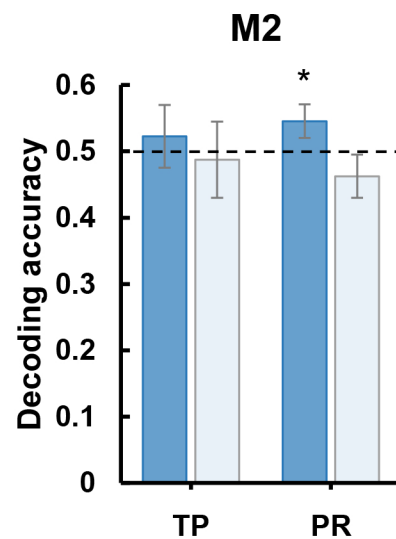

(F)

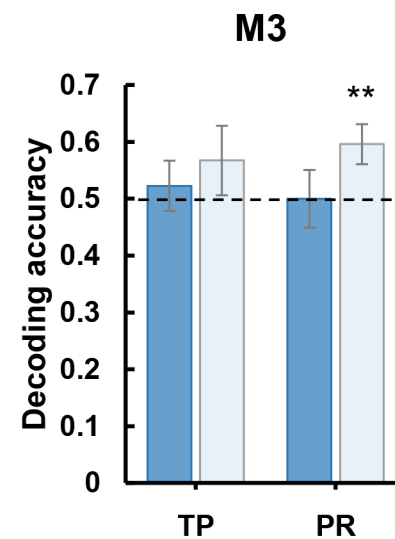

■ 1<sup>st</sup> half of sessions

□ 2<sup>nd</sup> half of sessions

Supplement: S18 Fig — (A–C) True-False color decoding accuracies in TP and PR in each monkey when combining all sessions, respectively. (D, E) True-false color decoding accuracies in TP and PR in each monkey based on the first and second halves of sessions, respectively. Bars display mean values +/− SEM. Black asterisks indicate a significant difference from the chance level (0.5, indicated by the dashed lines); *p < 0.05, **p < 0.01. The numbers above the bars indicate p-values that are marginally significant (p < 0.1). The data underlying this figure are available in S1 Data. (PDF) [file pbio.3002863.s018.pdf]

(A)

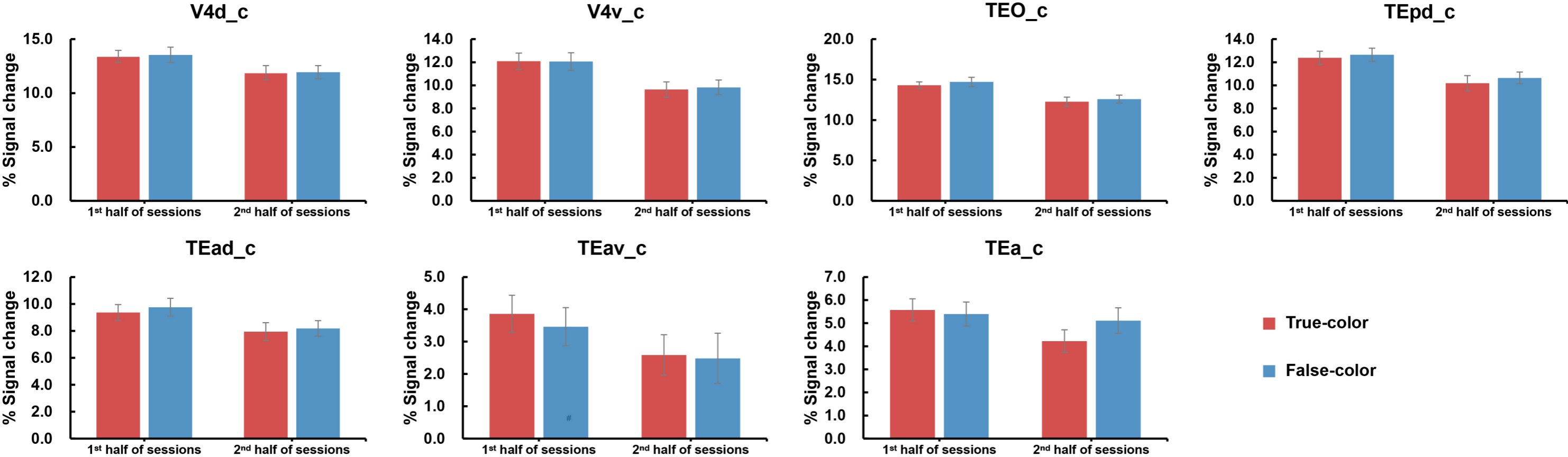

(B)

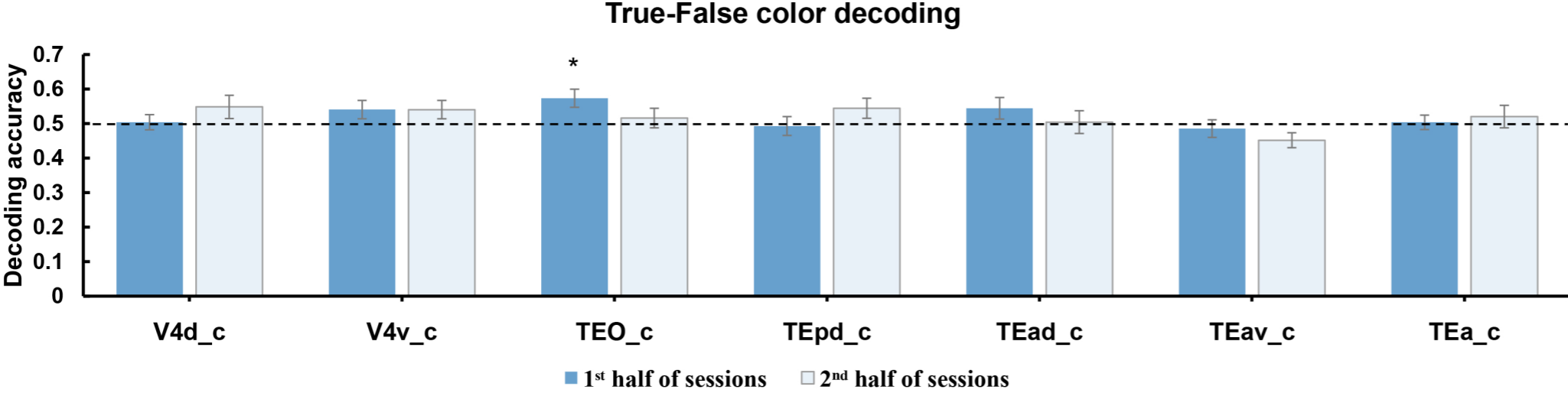

Supplement: S20 Fig — (A) Averaged fMRI responses to true- and false-colored objects in color patches across all three subjects for the first and second halves of sessions. (B) True-false color decoding accuracies across 3 monkeys for the first and second halves of sessions. Bars display mean values +/− SEM. Black asterisks indicate a significant difference from the chance level (0.5, indicated by the dashed lines); *q < 0.05. The data underlying this figure are available in S1 Data. (PDF) [file pbio.3002863.s020.pdf]

(A)

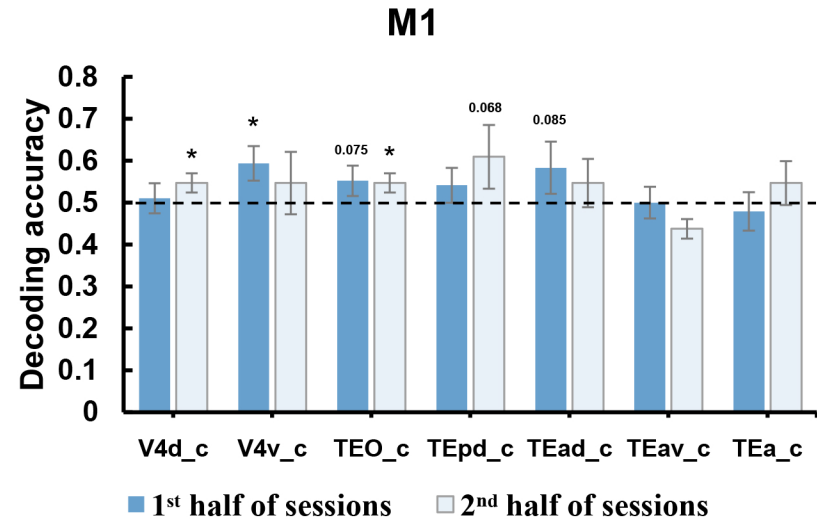

(B)

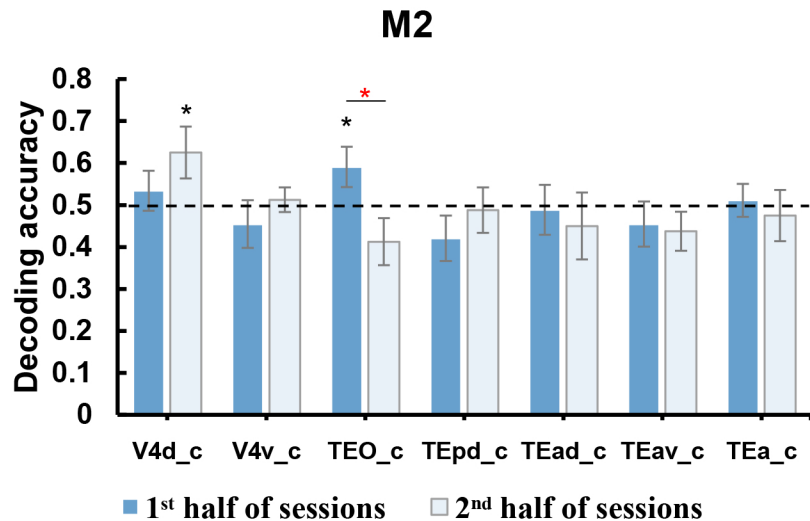

(C)

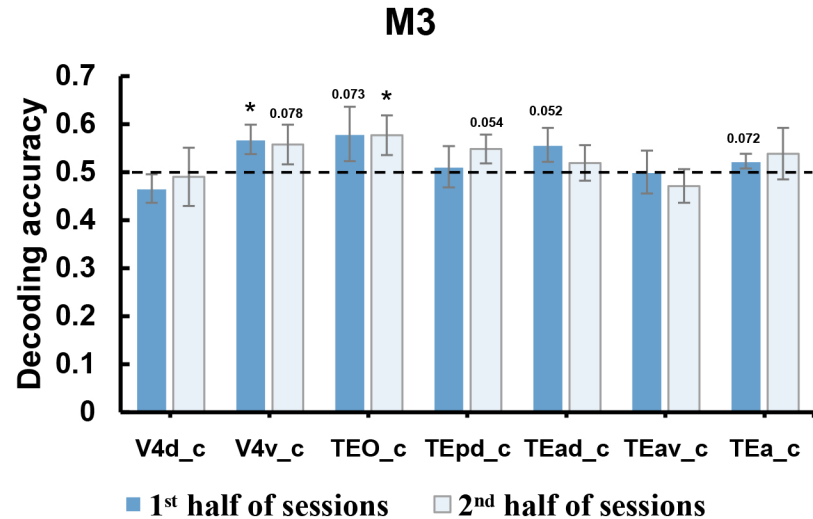

Supplement: S21 Fig — Bars display mean values +/− SEM. Black asterisks indicate a significant difference from the chance level (0.5, indicated by the dashed lines). Red asterisks indicate a significant difference between the first and second half of sessions; *p < 0.05. The numbers above the bars indicate p-values that are marginally significant (p < 0.1). The data underlying this figure are available in S1 Data. (PDF) [file pbio.3002863.s021.pdf]

## Amygdala

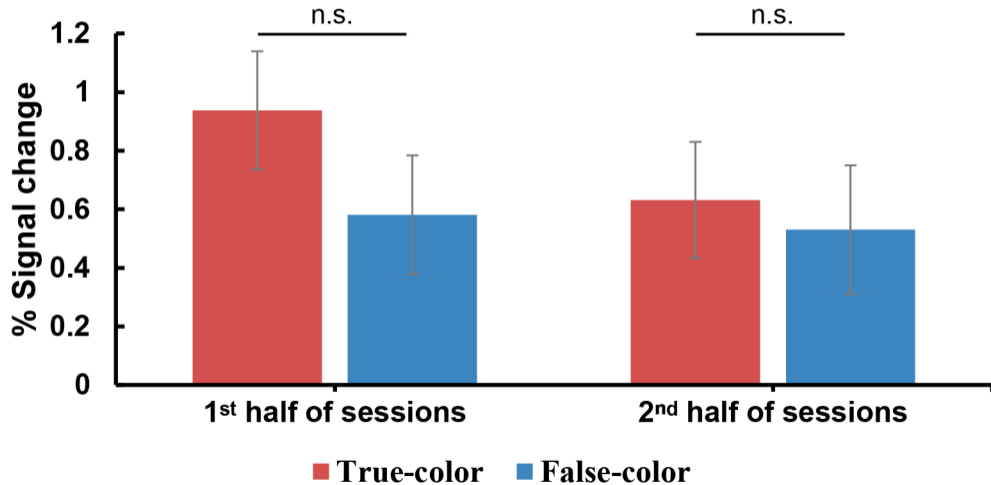

Supplement: S23 Fig — The main effect of true-false [F(1,126) = 2.348, p = 0.128] and the interaction effect between Period and true-false [F(1,126) = 0.733, p = 0.393] were not significant. Bars display mean values +/− SEM. n.s., not significant. The data underlying this figure are available in S1 Data. (PDF) [file pbio.3002863.s023.pdf]

(A)

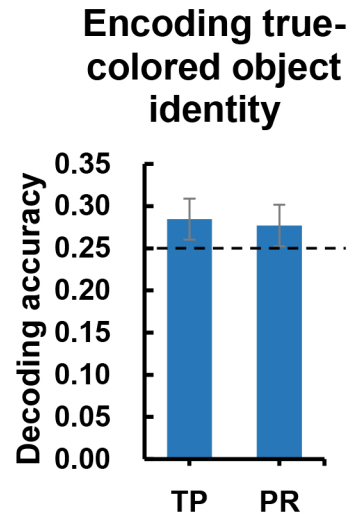

(B)

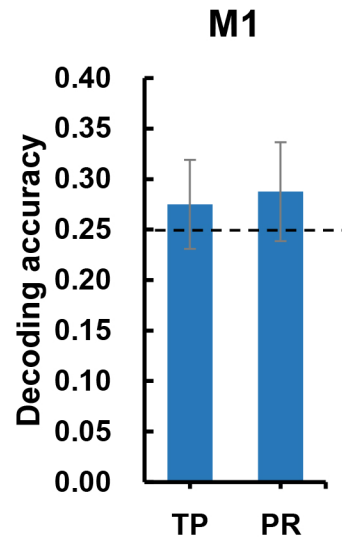

(C)

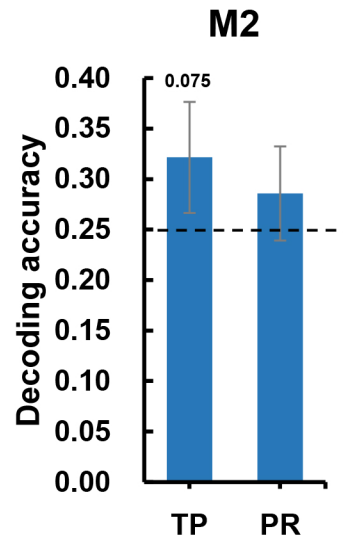

(D)

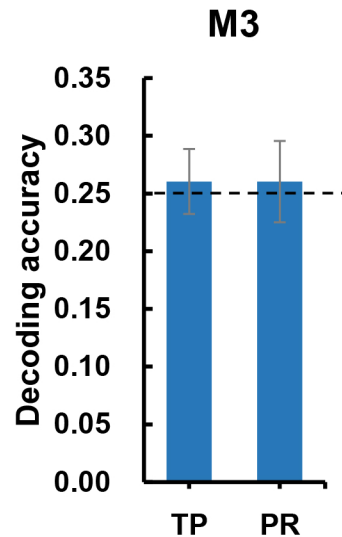

Supplement: S24 Fig — (A) Object identity encoding in TP and PR across 3 monkeys utilizing all true-colored objects in Exp 3. The group analysis yielded a trend but not significant decoding accuracy above the chance level for TP [F(1,128) = 2.281, p = 0.067, q = 0.126, one-tailed]. (B–D) Object identity encoding in TP and PR in each monkey, respectively. Bars display mean values +/− SEM. Dashed lines indicate the chance level (0.25). The numbers above the bars indicate p-values that are marginally significant (p < 0.1). The data underlying this figure are available in S1 Data. (PDF) [file pbio.3002863.s024.pdf]

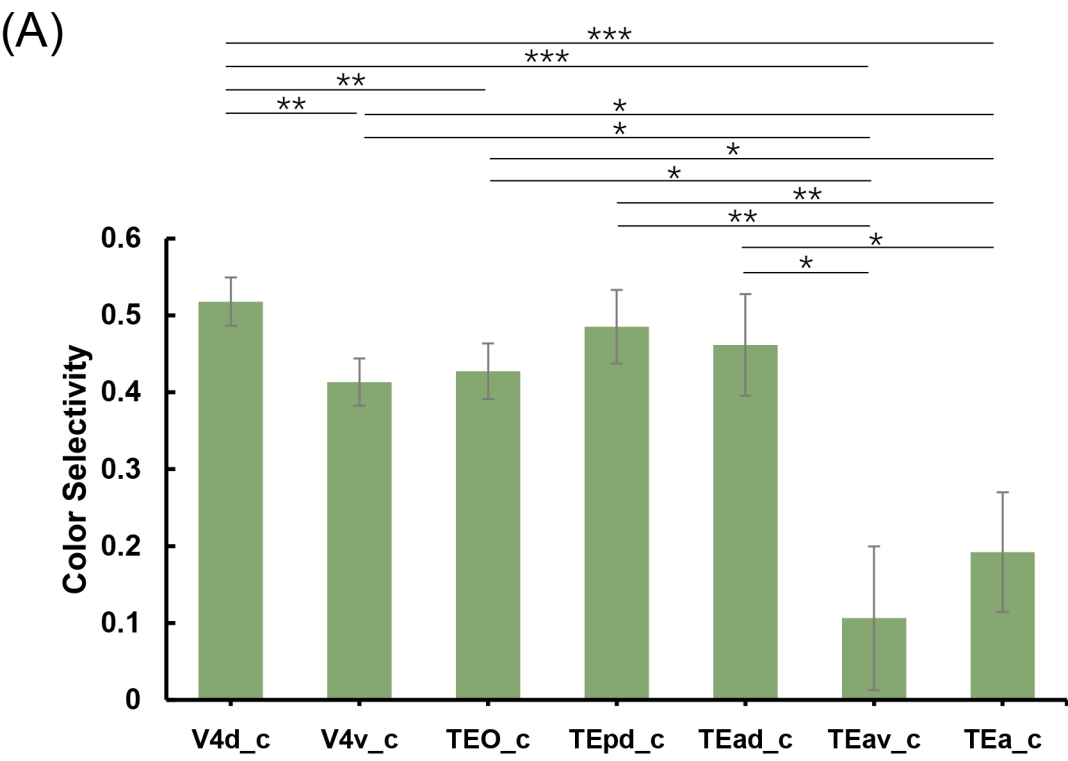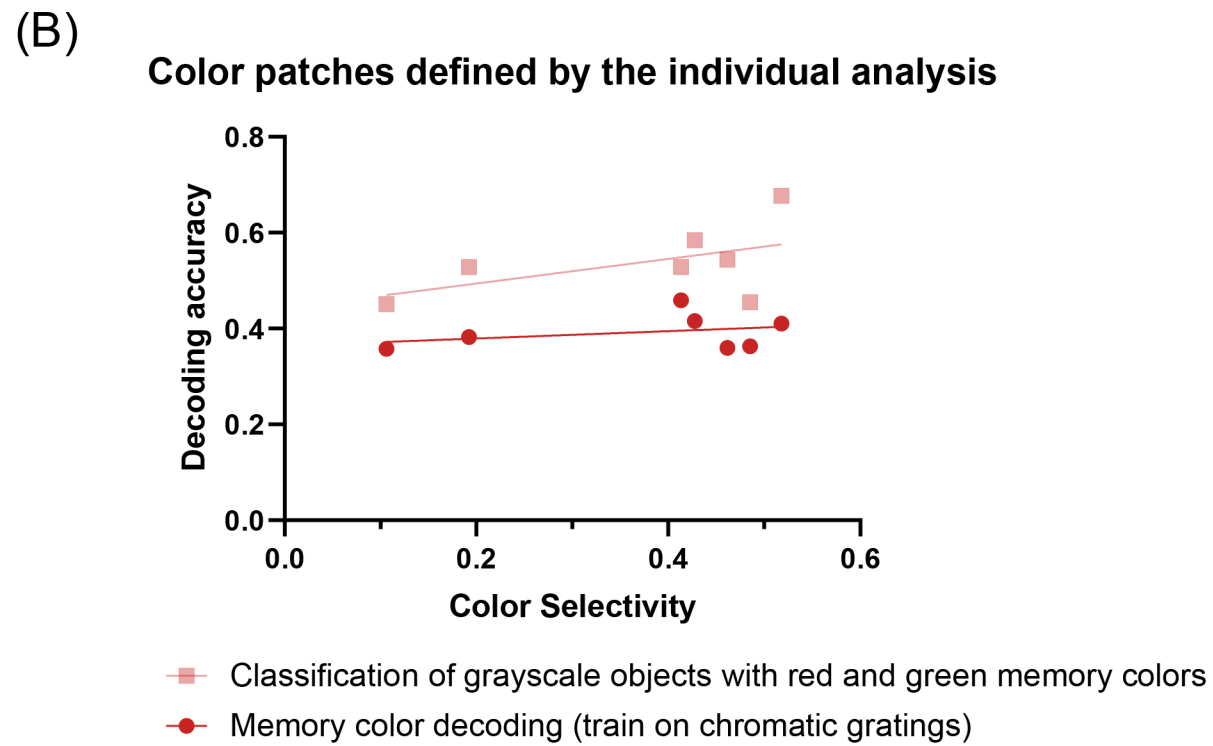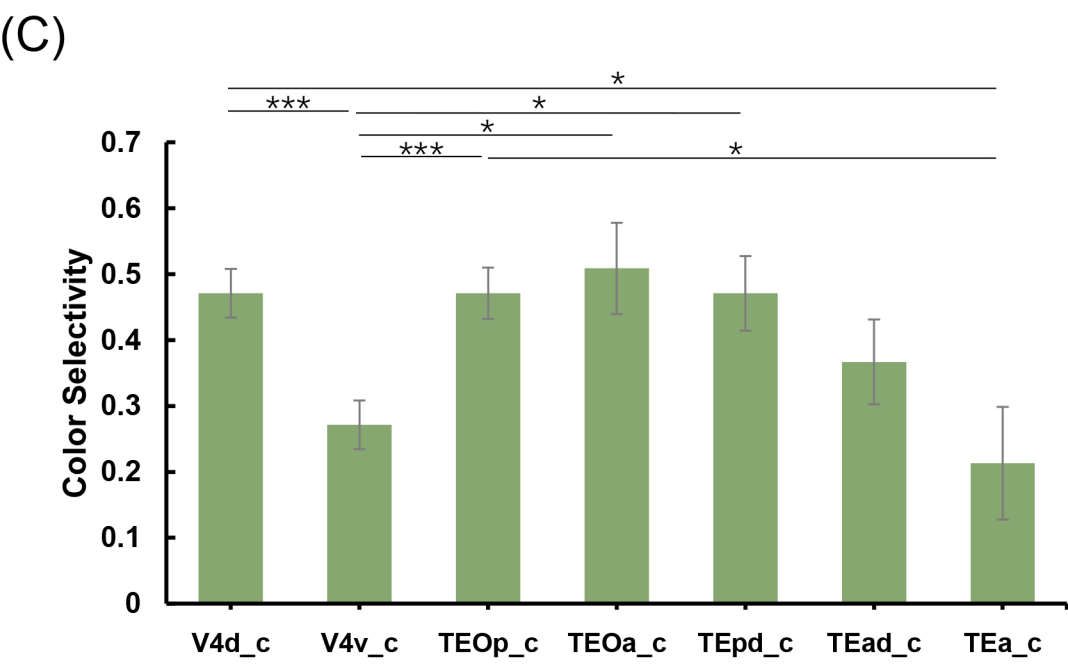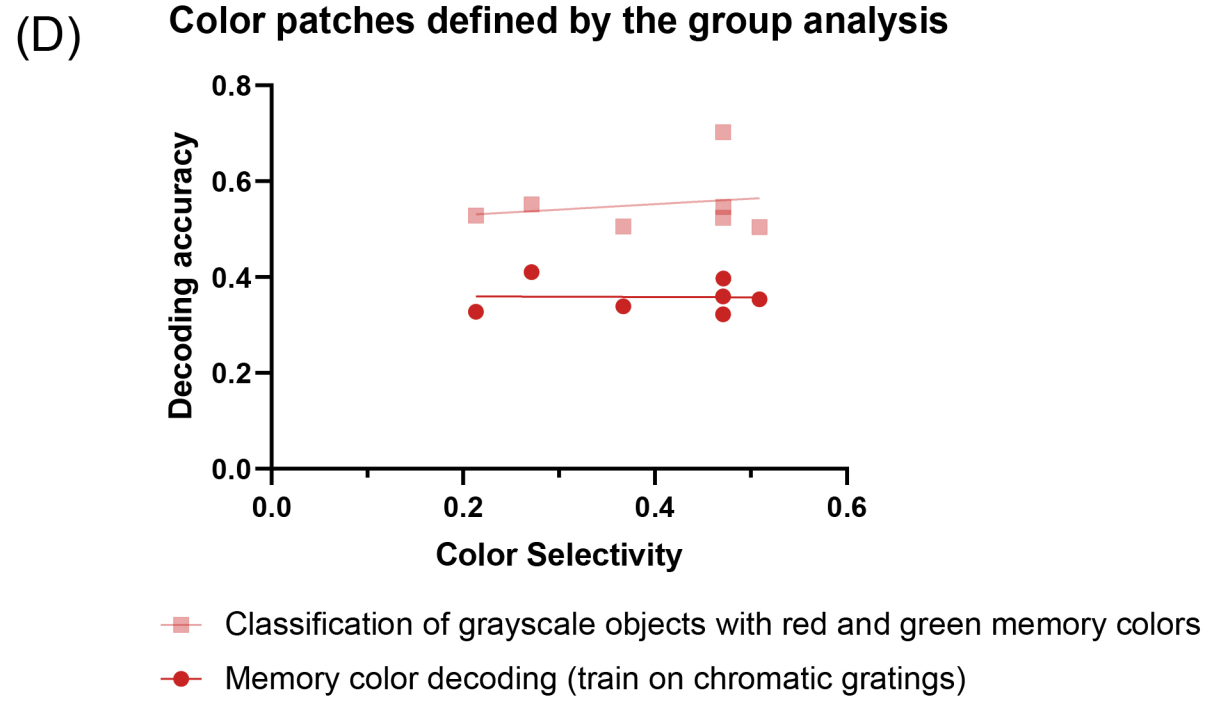

Supplement: S25 Fig — (A) Color selectivity in color patches defined by the individual analysis. (B) Correlations between the color selectivity and decoding accuracies of classification of grayscale objects with red and green memory colors (two-tailed Spearman correlation, r = 0.595, p = 0.170) and memory color decoding based on chromatic gratings training (two-tailed Spearman correlation, r = 0.179, p = 0.713) in color patches defined on the individual monkey’s activation map. (C) Color selectivity in color patches defined by the group analysis. (D) Correlations between the color selectivity and decoding accuracies of classification of grayscale objects with red and green memory colors (two-tailed Spearman correlation, r = −0.214, p = 0.662) and memory color decoding based on chromatic gratings training (two-tailed Spearman correlation, r = 0.214, p = 0.662) and in color patches defined by the group analysis. +q < 0.1, *q < 0.05, **q < 0.01, ***q < 0.001, Bonferroni corrected. The data underlying this figure are available in S1 Data. (PDF) [file pbio.3002863.s025.pdf]

**Combination 1**

**Combination 2**

**Combination 3**

**Combination 4**

**ROI  
Defining**

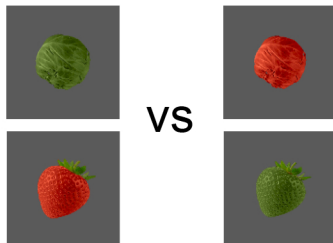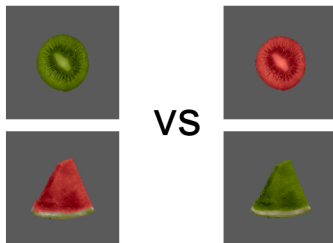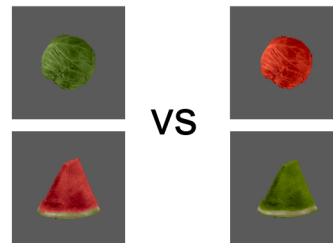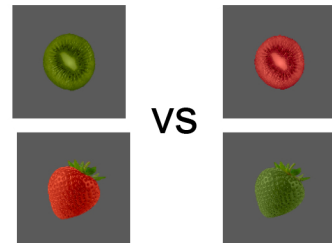

**ROI  
analyses**

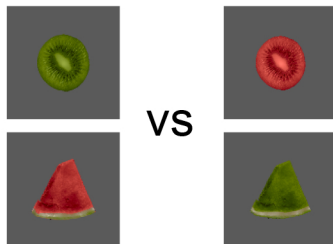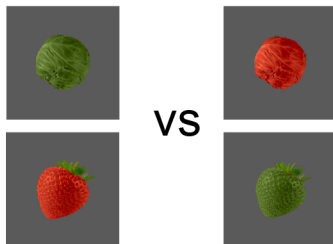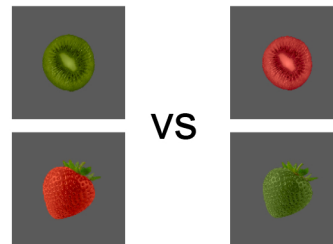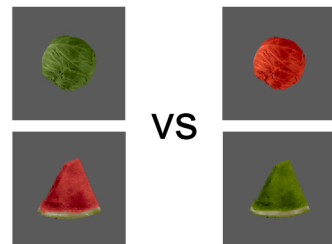

Supplement: S26 Fig — Half of the objects were used to define true-false ROIs in the ATL with the contrast of true-colored objects versus false-colored objects and then conducted the ROI analyses on the other half of the objects (e.g., in Combination 1, defining ROIs with true-colored cabbage and strawberry versus false-colored ones, then measuring responses on true- and false-colored kiwi and watermelon). (PDF) [file pbio.3002863.s026.pdf]
