# Supplementary material for: Object color knowledge representation occurs in the macaque brain despite the absence of a developed language system
Source: PLoS Biol. 2024 Oct 28;22(10):e3002863. doi: 10.1371/journal.pbio.3002863 (PMC11542842; doi:10.1371/journal.pbio.3002863)
Supplement: S4 Fig — (A–C) Averaged fMRI responses to 3 categories of color-diagnostic grayscale objects in color patches defined on individual activation maps (A) and by the group analysis (B) and in ATL (C) across all 3 monkeys. Bars display mean values +/− SEM. The data underlying this figure are available in S1 Data. (PDF) [file pbio.3002863.s004.pdf]

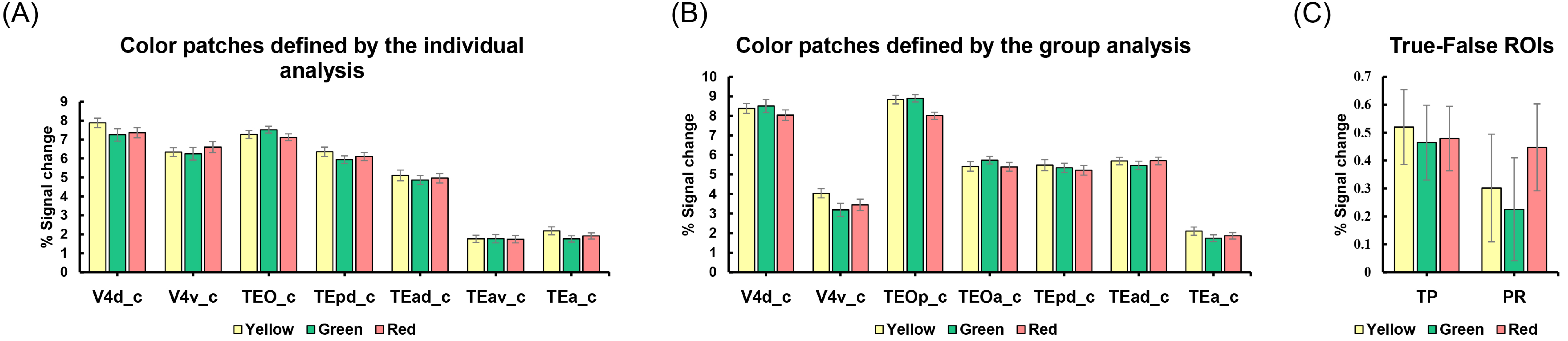
(A)
Color patches defined by the individual analysis
% Signal change
0
1
2
3
4
5
6
7
8
9
V4d_c
V4v_c
TEO_c
TEpd_c
TEad_c
TEav_c
TEa_c
Yellow
Green
Red
(B)
Color patches defined by the group analysis
% Signal change
0
1
2
3
4
5
6
7
8
9
10
V4d_c
V4v_c
TEOp_c
TEOa_c
TEpd_c
TEad_c
TEa_c
Yellow
Green
Red
(C)
True-False ROIs
% Signal change
0
0.1
0.2
0.3
0.4
0.5
0.6
0.7
TP
PR
Yellow
Green
Red
