# Supplementary material for: Object color knowledge representation occurs in the macaque brain despite the absence of a developed language system
Source: PLoS Biol. 2024 Oct 28;22(10):e3002863. doi: 10.1371/journal.pbio.3002863 (PMC11542842; doi:10.1371/journal.pbio.3002863)
Supplement: S5 Fig — (A) The results of classification of grayscale objects with 3 different memory colors: training the classifier to distinguish among the half of grayscale objects (e.g., strawberry, cabbage, and banana) and testing on the other half (e.g., watermelon slice, kiwi slice, and corn). Successful decoding was found in V4d_c, V4v_c, TEO_c, TEad_c, and TEa_c. (B) The “memory” color and shape similarities between training and test objects with red and green memory colors in various fold combinations. The second rows exhibited non-simultaneous alterations in “memory” color and shape similarity matrix values (framed by the red square), which were used to compute decoding accuracy rates in (C). For example, in Fold 1, we trained the classifier to distinguish the grayscale images of cabbage and watermelon and tested on kiwi and strawberry. In this case, kiwi might be correctly classified along with cabbage based on either color or shape, as for both properties, kiwi is more similar to cabbage than to watermelon. By contrast, strawberry is closer to watermelon in color but to cabbage in shape. Therefore, if a classifier identifies strawberries as more similar to watermelons with an accuracy significantly higher than chance, such a classifier could not be based on shape. (C) The results of the classification of grayscale objects with red and green memory colors in color patches based on the second rows of folds. Successful decoding was found in V4d_c, TEO_c, and TEad_c after excluding shape confound. Bars display mean values +/− SEM. Black asterisks indicate a significant difference from the chance level (0.333 in A, 0.5 in C, indicated by the dashed lines); *q < 0.05, **q < 0.01, ***q < 0.001. The data underlying this figure are available in S1 Data. (PDF) [file pbio.3002863.s005.pdf]

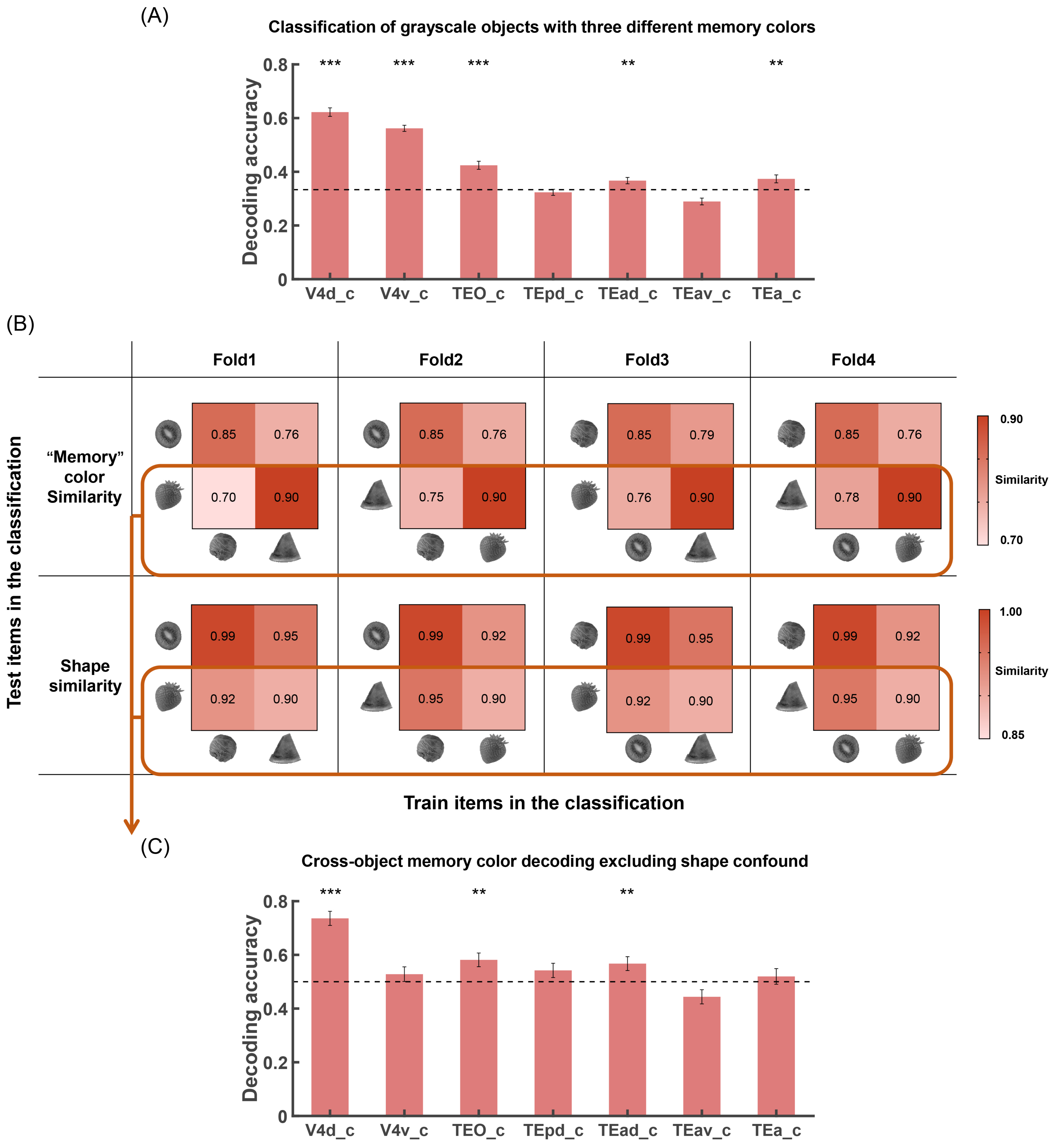
(A)
Classification of grayscale objects with three different memory colors
Decoding accuracy
0.8
0.6
0.4
0.2
0
*** *** *** ** **
V4d_c V4v_c TEO_c TEpd_c TEad_c TEav_c TEa_c
(B)
Fold1 Fold2 Fold3 Fold4
"Memory" color Similarity
0.85 0.76 0.70 0.90
0.85 0.76 0.75 0.90
0.85 0.79 0.76 0.90
0.85 0.76 0.78 0.90
0.90 Similarity 0.70
Shape similarity
0.99 0.95 0.92 0.90
0.99 0.92 0.95 0.90
0.99 0.95 0.92 0.90
0.99 0.92 0.95 0.90
1.00 Similarity 0.85
Test items in the classification
Train items in the classification
(C)
Cross-object memory color decoding excluding shape confound
Decoding accuracy
0.8
0.6
0.4
0.2
0
*** ** **
V4d_c V4v_c TEO_c TEpd_c TEad_c TEav_c TEa_c
