# Supplementary material for: Object color knowledge representation occurs in the macaque brain despite the absence of a developed language system
Source: PLoS Biol. 2024 Oct 28;22(10):e3002863. doi: 10.1371/journal.pbio.3002863 (PMC11542842; doi:10.1371/journal.pbio.3002863)
Supplement: S9 Fig — (A) Results of classification of grayscale objects with red and green memory colors: training the classifier to distinguish half set of the red and green color-diagnostic grayscale objects and testing on the other half in Exp 2. (B) Results of memory color decoding based on chromatic gratings training: training the classifier to distinguish among 3 chromatic gratings in Exp 1 and then testing on 3 categories of grayscale objects in Exp 2. Bars display mean values +/− SEM. Black asterisks indicate a significant difference from the chance level (0.5 in A, 0.333 in B, indicated by the dashed lines); *p < 0.05. The data underlying this figure are available in S1 Data. (PDF) [file pbio.3002863.s009.pdf]

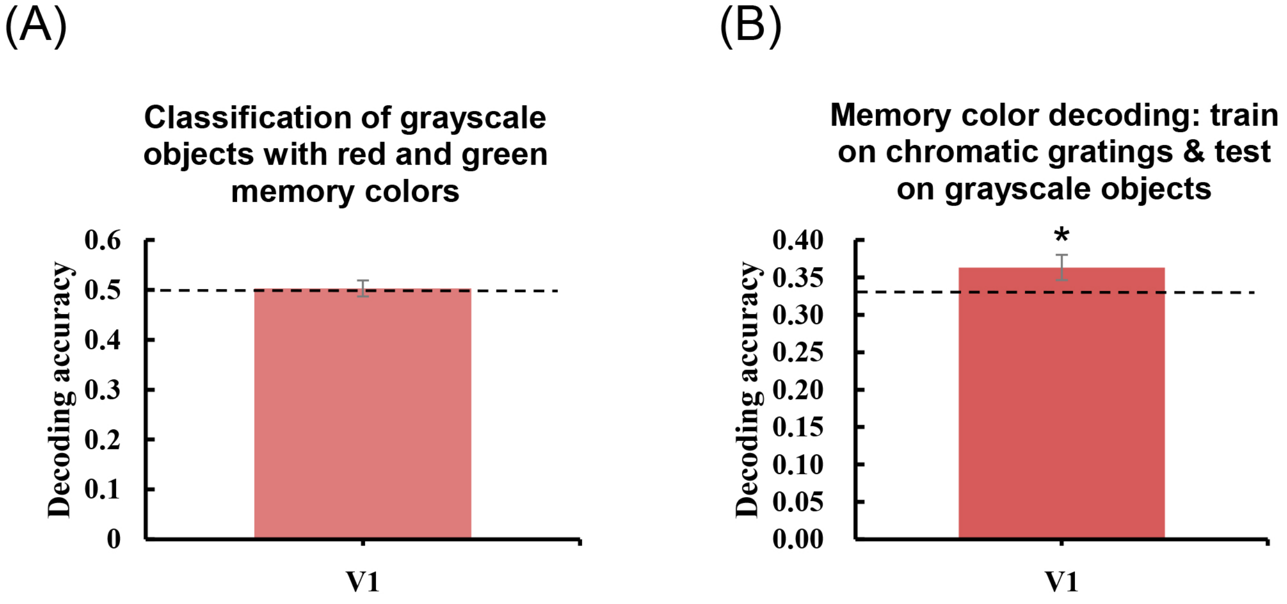
(A)
Classification of grayscale objects with red and green memory colors
Decoding accuracy
0.6
0.5
0.4
0.3
0.2
0.1
0
V1
(B)
Memory color decoding: train on chromatic gratings & test on grayscale objects
*
Decoding accuracy
0.40
0.35
0.30
0.25
0.20
0.15
0.10
0.05
0.00
V1
