# Supplementary material for: Object color knowledge representation occurs in the macaque brain despite the absence of a developed language system
Source: PLoS Biol. 2024 Oct 28;22(10):e3002863. doi: 10.1371/journal.pbio.3002863 (PMC11542842; doi:10.1371/journal.pbio.3002863)
Supplement: S13 Fig — (A) The true-color versus false-color in PR are shown in the coronal slices for each of the 3 subjects (M1 to M3). The threshold for each cluster is listed on the right side of the slices. (B, C) The true-color versus false-color in the first part of sessions (B), and the second part of sessions (C) in PR are shown in the coronal slices for each of the 3 subjects (M1 to M3) at p < 0.05, respectively. Each slice’s anterior/posterior position is indicated on the top left corner (mm relative to the interaural canal). (D) Averaged fMRI responses to true- and false-colored objects in PR across all 3 subjects based on all the sessions [F(1,128) = 0.050, q = 0.823, η2 < 0.001; two-tailed]. (E) Averaged fMRI responses to true- and false-colored objects in PR across all 3 subjects from the first and second halves of sessions [the interaction effect between Period (first half versus second half) and True-False across 3 monkeys: F(1,126) = 0.305, q = 0.582, η2 = 0.002]. The data underlying this figure are available in S1 Data. (PDF) [file pbio.3002863.s013.pdf]

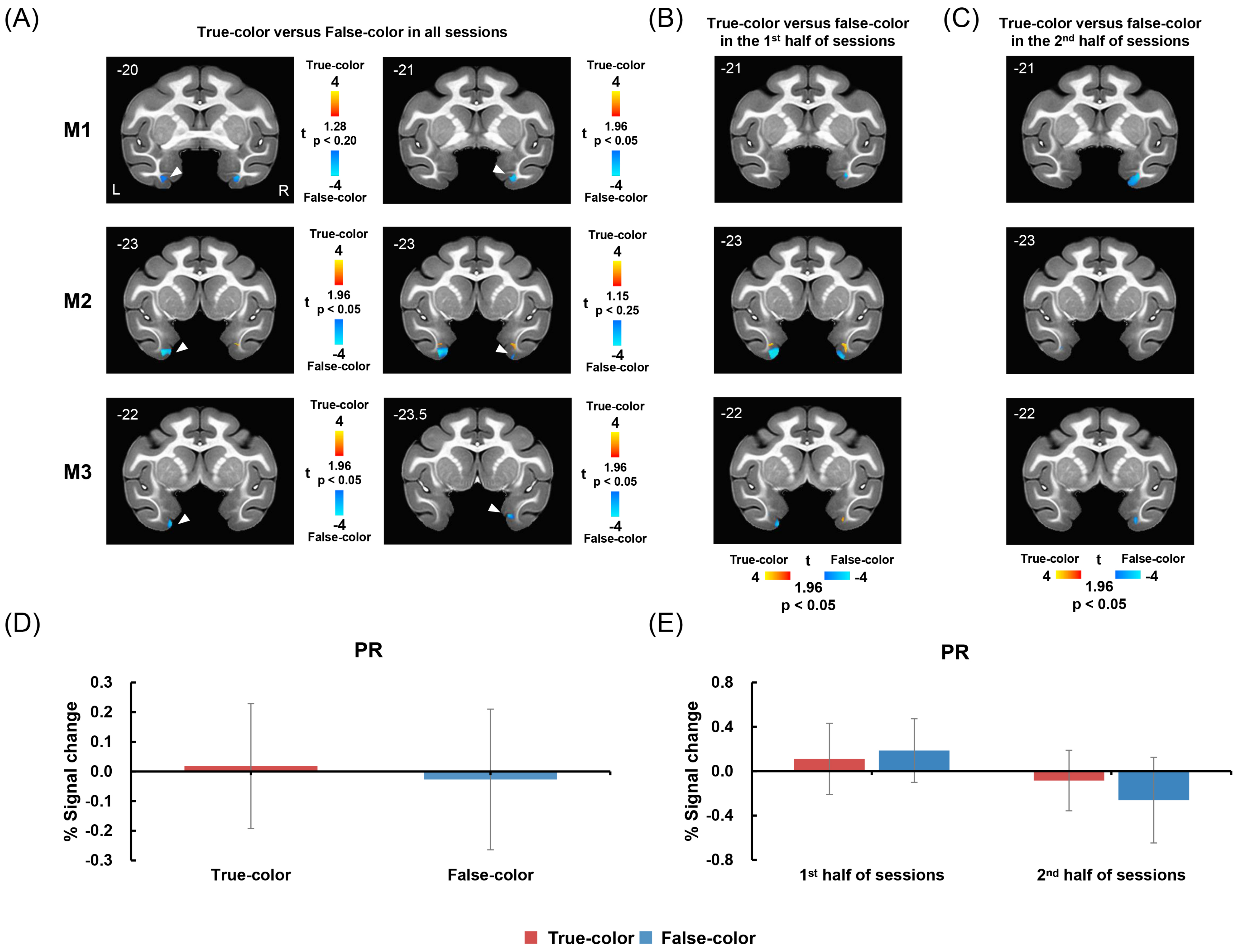
(A)
True-color versus False-color in all sessions
M1
M2
M3
-20
-21
-23
-23
-22
-23.5
L
R
True-color
4
t
1.28
p < 0.20
-4
False-color
1.96
p < 0.05
1.15
p < 0.25
(B)
True-color versus false-color in the 1st half of sessions
(C)
True-color versus false-color in the 2nd half of sessions
True-color t False-color
4 1.96 -4
p < 0.05
(D)
PR
% Signal change
0.3
0.2
0.1
0.0
-0.1
-0.2
-0.3
True-color
False-color
(E)
PR
0.8
0.4
0.0
-0.4
-0.8
1st half of sessions
2nd half of sessions
True-color False-color
