# Supplementary material for: Object color knowledge representation occurs in the macaque brain despite the absence of a developed language system
Source: PLoS Biol. 2024 Oct 28;22(10):e3002863. doi: 10.1371/journal.pbio.3002863 (PMC11542842; doi:10.1371/journal.pbio.3002863)
Supplement: S15 Fig — (A) The conjunction map that combined the true-color versus false-color contrast with the interaction effect between Period (first half versus second half of sessions) and True-False for each of the 3 subjects (M1 to M3) at p < 0.01. Each slice’s anterior/posterior position is indicated on the top left corner (mm relative to the interaural canal). (B–D) FMRI responses to true- and false-colored objects in TP from the first and second halves of sessions for each subject, respectively. Individual analyses demonstrated similar trends for the 3 monkeys, although the results were not significant, likely due to the limited sample size used as a result of the request to avoid double dipping and the impact of the learning effect. (E–G) fMRI responses to true- and false-colored objects in TP for each session in each subject. Bars display mean values +/− SEM. Red asterisks indicate a significant difference between responses evoked by true- and false-colored stimuli; *p < 0.05, **p < 0.01, ***p < 0.001. The data underlying this figure are available in S1 Data. (PDF) [file pbio.3002863.s015.pdf]

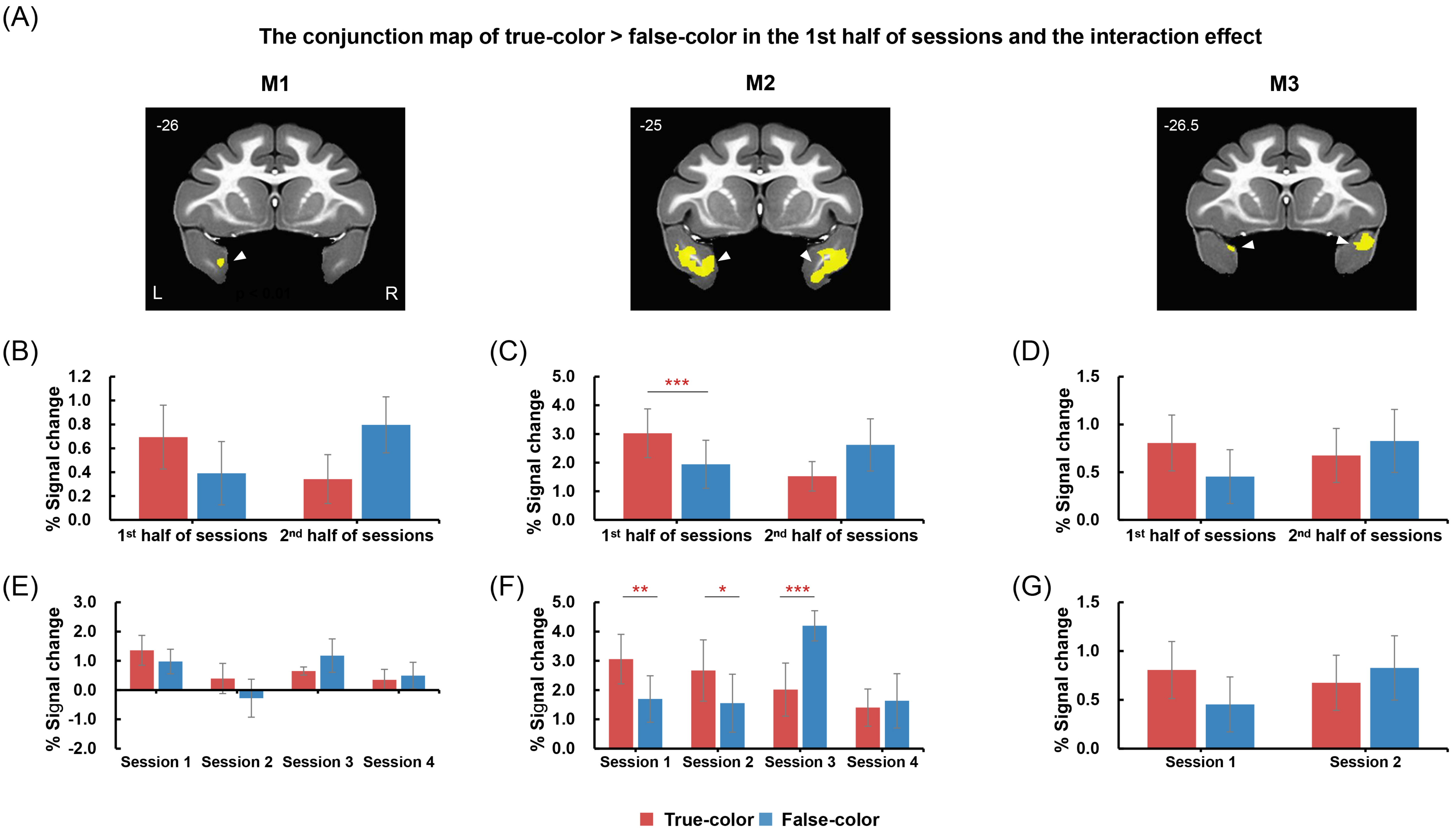

(A)
The conjunction map of true-color > false-color in the 1st half of sessions and the interaction effect
M1
M2
M3
-26
-25
-26.5
L
R
(B)
(C)
(D)
(E)
(F)
(G)
% Signal change
1st half of sessions
2nd half of sessions
Session 1
Session 2
Session 3
Session 4
***
**
*
True-color
False-color
