# Supplementary material for: Object color knowledge representation occurs in the macaque brain despite the absence of a developed language system
Source: PLoS Biol. 2024 Oct 28;22(10):e3002863. doi: 10.1371/journal.pbio.3002863 (PMC11542842; doi:10.1371/journal.pbio.3002863)
Supplement: S19 Fig — (A) True-False color decoding accuracies in TP and PR across 3 monkeys based on the first and second halves of sessions. (B) True-False color decoding accuracies across 3 monkeys when combining all sessions. Bars display mean values +/− SEM. Black asterisks indicate a significant difference from the chance level (0.5, indicated by the dashed lines); *q < 0.05. The data underlying this figure are available in S1 Data. (PDF) [file pbio.3002863.s019.pdf]

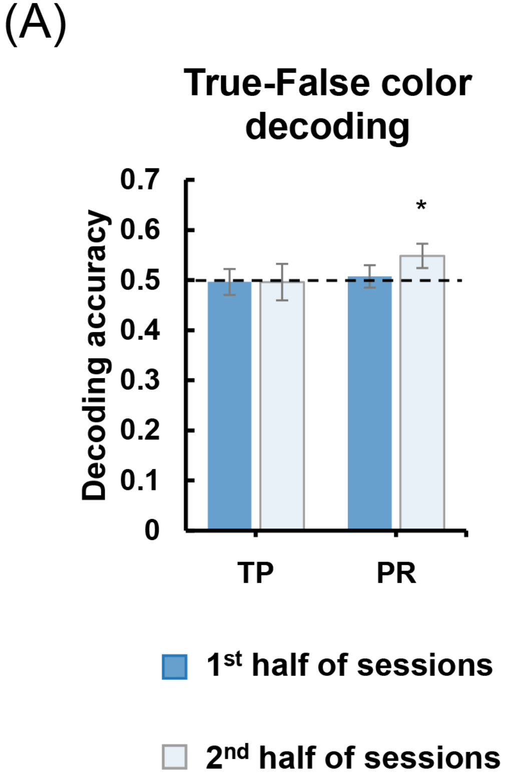
(A)
True-False color decoding
Decoding accuracy
0.7
0.6
0.5
0.4
0.3
0.2
0.1
0
*
TP
PR
(B)
True-False color decoding
Decoding accuracy
0.6
0.5
0.4
0.3
0.2
0.1
0
TP
PR
1st half of sessions
2nd half of sessions
