# Supplementary material for: Object color knowledge representation occurs in the macaque brain despite the absence of a developed language system
Source: PLoS Biol. 2024 Oct 28;22(10):e3002863. doi: 10.1371/journal.pbio.3002863 (PMC11542842; doi:10.1371/journal.pbio.3002863)
Supplement: S22 Fig — (A) Results of classification of grayscale objects with red and green memory colors in each monkey: training the classifier to distinguish half set of the red and green color-diagnostic grayscale objects and testing on the other half in Exp 2. (B) Results of memory color decoding based on chromatic gratings training in each monkey: training the classifier to distinguish among three chromatic gratings in Exp 1 and then testing on 3 categories of grayscale objects in Exp 2. Bars display mean values +/− SEM. Dashed lines indicate the chance level (0.5 in A and 0.333 in B); *p < 0.05. The numbers above the bars indicate p-values that are marginally significant (p < 0.1). The data underlying this figure are available in S1 Data. (PDF) [file pbio.3002863.s022.pdf]

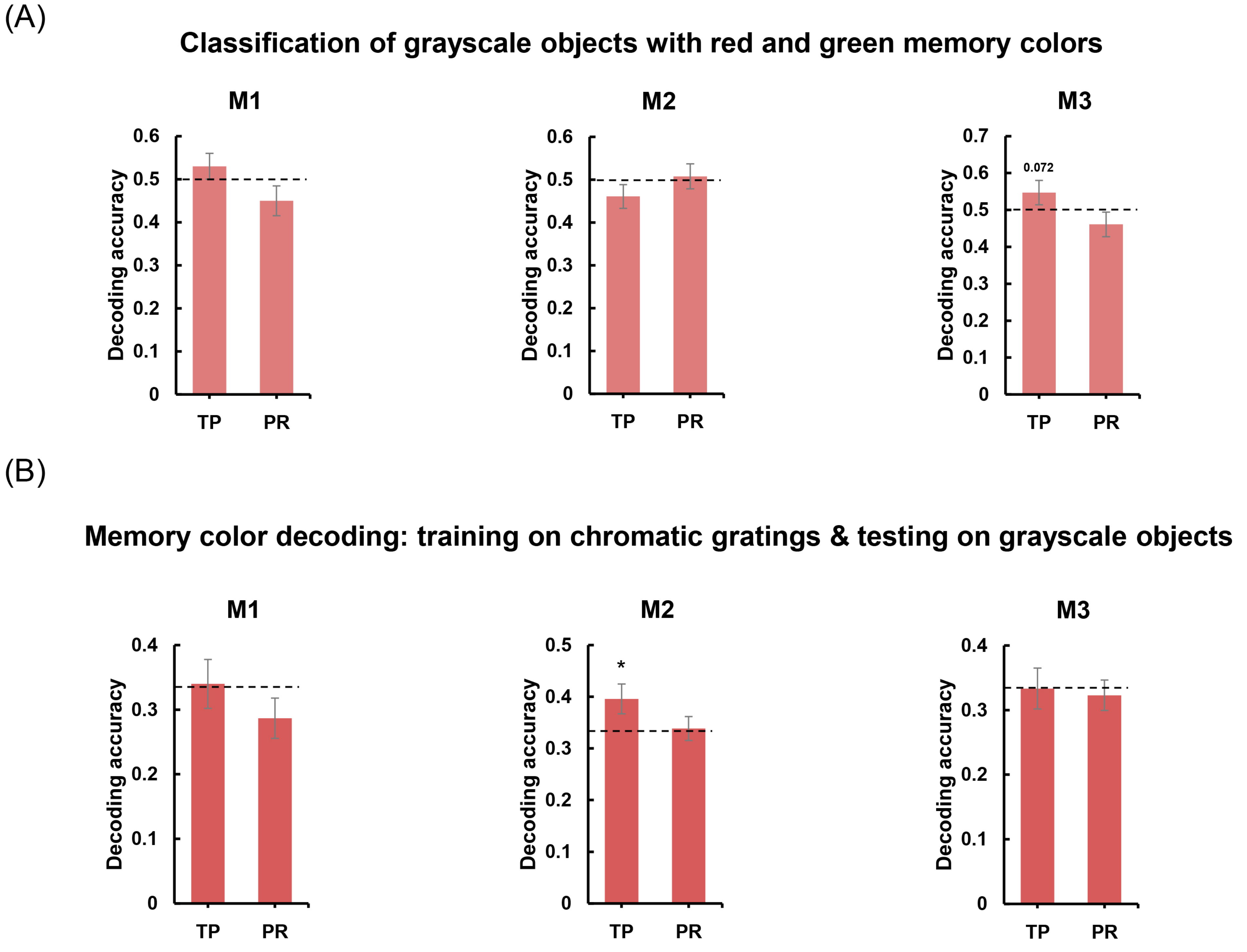
(A)
Classification of grayscale objects with red and green memory colors
M1
M2
M3
0.072
Decoding accuracy
TP
PR
(B)
Memory color decoding: training on chromatic gratings & testing on grayscale objects
M1
M2
M3
*
Decoding accuracy
TP
PR
