# Supplementary material for: Object color knowledge representation occurs in the macaque brain despite the absence of a developed language system
Source: PLoS Biol. 2024 Oct 28;22(10):e3002863. doi: 10.1371/journal.pbio.3002863 (PMC11542842; doi:10.1371/journal.pbio.3002863)
Supplement: S27 Fig — (A) Results of classification of grayscale objects with red and green memory colors: training the classifier to distinguish half set of the red and green color-diagnostic grayscale objects and testing on the other half in Exp 2. (B) Results of memory color decoding based on chromatic gratings training: training the classifier to distinguish among 3 chromatic gratings in Exp 1 and then testing on 3 categories of grayscale objects in Exp 2. (C) Results of true-false color decoding: training on true- and false-colored objects in N-1 runs and testing on the left-out run in Exp 3 when combining all sessions, in the first half of sessions and in the second half of sessions. (D) Results of object identity decoding: training on all true-colored objects in N-1 runs and testing on the left-out run when combining all sessions in Exp 3. Bars display mean values +/− SEM. Dashed lines indicate the chance level (0.5 in A and C; 0.333 in B; 0.25 in D); *q < 0.05. The data underlying this figure are available in S1 Data. (PDF) [file pbio.3002863.s027.pdf]

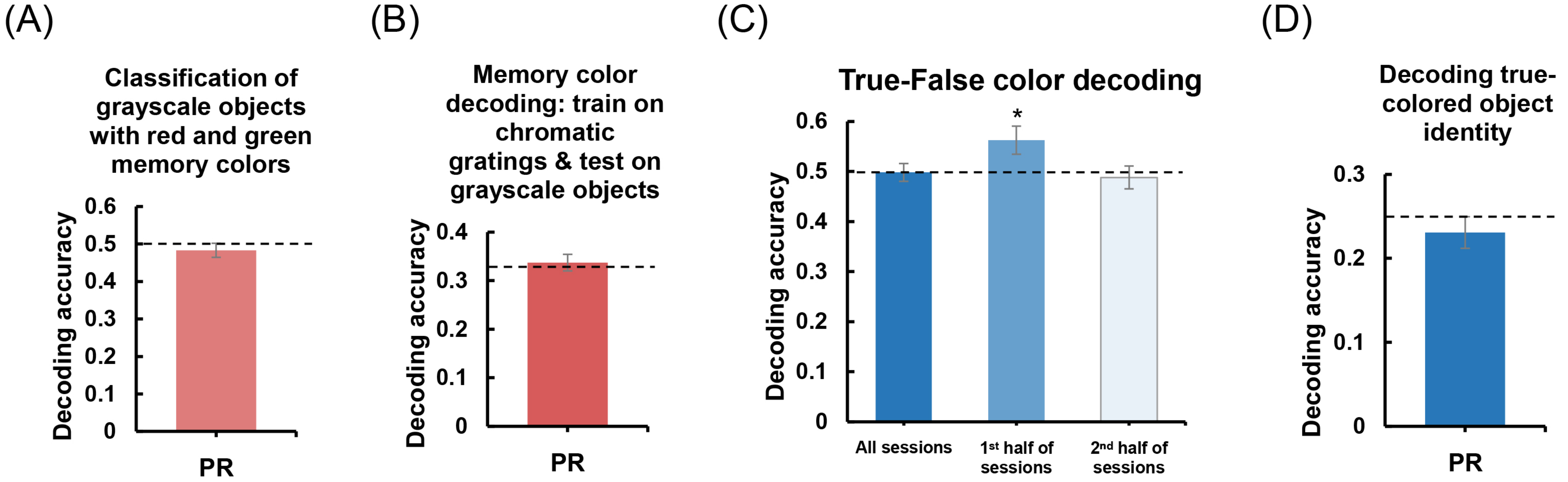
(A)
Classification of grayscale objects with red and green memory colors
Decoding accuracy
0.6
0.5
0.4
0.3
0.2
0.1
0
PR
(B)
Memory color decoding: train on chromatic gratings & test on grayscale objects
Decoding accuracy
0.4
0.3
0.2
0.1
0
PR
(C)
True-False color decoding
Decoding accuracy
0.6
0.5
0.4
0.3
0.2
0.1
0
*
All sessions
1st half of sessions
2nd half of sessions
(D)
Decoding true-colored object identity
Decoding accuracy
0.3
0.2
0.1
0
PR
