# Supplementary material for: Object color knowledge representation occurs in the macaque brain despite the absence of a developed language system
Source: PLoS Biol. 2024 Oct 28;22(10):e3002863. doi: 10.1371/journal.pbio.3002863 (PMC11542842; doi:10.1371/journal.pbio.3002863)
Supplement: S1 Table — (DOCX) [file pbio.3002863.s028.docx]

**S1 Table. The correspondence between names of color patches in the present study and previous studies (1).**

| **Original Names of Color Patches** | **Revised Names of Color Patches Based on the Individual Analysis** | **Revised Names of Color Patches Based on the Group Analysis** |
| --- | --- | --- |
| PITd | V4d_c | V4d_c |
| V4v | V4v_c | V4v_c |
| PLc | TEO_c | TEOp_c, TEOa_c |
| CLc | TEpd_c | TEpd_c |
| ALc | TEad_c | TEad_c |
| AVc | TEav_c | - |
| AFc | TEa_c | TEa_c |

1. Lafer-Sousa R, Conway BR. Parallel, multi-stage processing of colors, faces and shapes in macaque inferior temporal cortex. Nat Neurosci. 2013;16(12):1870-8.
