# Supplementary material for: Object color knowledge representation occurs in the macaque brain despite the absence of a developed language system
Source: PLoS Biol. 2024 Oct 28;22(10):e3002863. doi: 10.1371/journal.pbio.3002863 (PMC11542842; doi:10.1371/journal.pbio.3002863)
Supplement: S2 Table — (DOCX) [file pbio.3002863.s029.docx]

**S2 Table. GLMM results of main effects of True-False in color patches.**

|  | **F value** | **t value** | **p value** | **q value** | **Cohen's d** |
| --- | --- | --- | --- | --- | --- |
| **V4d_c** | 0.122 | -0.349 | 0.727 | 0.731 | -0.062 |
| **V4v_c** | 0.119 | -0.344 | 0.731 | 0.731 | -0.061 |
| **TEO_c** | 2.607 | -1.615 | 0.109 | 0.499 | -0.285 |
| **TEpd_c** | 1.558 | -1.248 | 0.214 | 0.499 | -0.221 |
| **TEad_c** | 1.788 | -1.337 | 0.183 | 0.499 | -0.236 |
| **TEav_c** | 0.539 | 0.734 | 0.464 | 0.650 | 0.130 |
| **TEa_c** | 1.026 | -1.013 | 0.313 | 0.548 | -0.179 |
