# Supplementary material for: Object color knowledge representation occurs in the macaque brain despite the absence of a developed language system
Source: PLoS Biol. 2024 Oct 28;22(10):e3002863. doi: 10.1371/journal.pbio.3002863 (PMC11542842; doi:10.1371/journal.pbio.3002863)
Supplement: S3 Table — (DOCX) [file pbio.3002863.s030.docx]

**S3 Table. GLMM results of fMRI responses to true- and false-colored objects in TP and PR for each subject.**

|  |  | **1^st^** | | | | **2^nd^** | | | | **Interaction effect** | | |
| --- | --- | --- | --- | --- | --- | --- | --- | --- | --- | --- | --- | --- |
|  |  | **F value** | **t value** | **p value** | **Cohen's d** | **F value** | **t value** | **p value** | **Cohen's d** | **F value** | **p value** | **η2** |
| **TP** | **M1** | 2.437 | 1.561 | 0.127 | 0.527 | 1.069 | -1.034 | 0.308 | -0.350 | 3.392 | 0.074 | 0.088 |
|  | **M2** | 14.644 | 3.827 | <0.001 | 4.751 | 1.034 | -1.017 | 0.316 | -0.330 | 5.502 | 0.024 | 0.127 |
|  | **M3** | 0.712 | 0.844 | 0.403 | 0.215 | 1.662 | -1.289 | 0.204 | -0.389 | 2.114 | 0.153 | 0.046 |
| **PR** | **M1** | 2.759 | 1.661 | 0.106 | 0.933 | <0.001 | -0.012 | 0.991 | -0.004 | 0.774 | 0.385 | 0.022 |
|  | **M2** | 3.972 | -1.993 | 0.053 | -0.647 | 12.702 | 3.564 | 0.001 | 1.156 | 12.964 | 0.001 | 0.254 |
|  | **M3** | 0.028 | 0.167 | 0.868 | 0.050 | 0.256 | -0.505 | 0.616 | -0.152 | 0.265 | 0.609 | 0.006 |
