# Supplementary material for: Object color knowledge representation occurs in the macaque brain despite the absence of a developed language system
Source: PLoS Biol. 2024 Oct 28;22(10):e3002863. doi: 10.1371/journal.pbio.3002863 (PMC11542842; doi:10.1371/journal.pbio.3002863)
Supplement: S4 Table — (DOCX) [file pbio.3002863.s031.docx]

**S4 Table. GLMM results of interaction effects between Period and True-False across three monkeys in color patches.**

|  | **F value** | **p value** | **q value** | **η2** |
| --- | --- | --- | --- | --- |
| **V4d_c** | 0.030 | 0.863 | 0.972 | <0.001 |
| **V4v_c** | 0.257 | 0.613 | 0.972 | 0.002 |
| **TEO_c** | 0.001 | 0.972 | 0.972 | <0.001 |
| **TEpd_c** | 0.107 | 0.744 | 0.972 | <0.001 |
| **TEad_c** | 0.108 | 0.742 | 0.972 | <0.001 |
| **TEav_c** | 0.166 | 0.685 | 0.972 | 0.001 |
| **TEa_c** | 2.716 | 0.102 | 0.714 | 0.021 |
