# Supplementary material for: Object color knowledge representation occurs in the macaque brain despite the absence of a developed language system
Source: PLoS Biol. 2024 Oct 28;22(10):e3002863. doi: 10.1371/journal.pbio.3002863 (PMC11542842; doi:10.1371/journal.pbio.3002863)
Supplement: S5 Table — (DOCX) [file pbio.3002863.s032.docx]

**S5 Table. Parameters of chromatic and achromatic stimuli used in Exp 1.**

|  | **Hue (°)** | **Foreground** | | **Background** | |
| --- | --- | --- | --- | --- | --- |
|  |  | **RGB** | **CIE1931（x,y,L）** | **RGB** | **CIE1931（x,y,L）** |
| **Yellow** | 60 | (86.24,86.24,0) | (0.45,0.51,14.55) | (100.14,100.14,100.14) | (0.37,0.39,14.55) |
| **Green** | 120 | (0,112.65,0) | (0.39,0.58,14.55) | (100.14,100.14,100.14) | (0.37,0.39,14.55) |
| **Red** | 0 | (228.11,0,0) | (0.65,0.34,14.55) | (100.14,100.14,100.14) | (0.37,0.39,14.55) |
| **25%** | - | (110.38,110.38,110.38) | (0.37,0.39,18.19) | (88.53,88.53,88.53) | (0.37,0.39,10.91) |
| **50%** | - | (119.64,119.64,119.64) | (0.37,0.39,21.83) | (74.83,74.83,74.83) | (0.37,0.39,7.28) |
| **75%** | - | (128.14,128.14,128.14) | (0.37,0.39,25.46) | (57.39,57.39,57.39) | (0.37,0.39,3.64) |
