# Supplementary material for: Object color knowledge representation occurs in the macaque brain despite the absence of a developed language system
Source: PLoS Biol. 2024 Oct 28;22(10):e3002863. doi: 10.1371/journal.pbio.3002863 (PMC11542842; doi:10.1371/journal.pbio.3002863)
Supplement: S1 Text — (DOCX) [file pbio.3002863.s033.docx]

**Behavioral Experiment**

**Design and Task**

In consideration of the well-being of the animals, one of the tested individuals (M1) was unable to participate in any further experiments and was put on rest forever. Consequently, we conducted the behavioral tests using two subjects, namely M2 and M3. These subjects were trained to perform a free-viewing visual paired comparison (VPC) task, as detailed in our previous study (1). In brief, the eye positions of the monkeys were monitored using an infrared pupil tracking system (ISCAN, Inc., Woburn, MA, USA) with a sampling rate of 120 Hz. Each trial consisted of three periods: fixation, free viewing, and reward following correct responses or a time-out period after errors (S1A Fig). After the monkey fixated on the red triangle fixation spot for 500 ms, the fixation spot disappeared, and a pair of images (~6°for each image and 4.5 degrees away from the center of the screen) appeared on the screen and remained visible for up to 4 s. During this time, the subjects were allowed to freely look at both images. Subsequently, the images disappeared, and the subjects were rewarded with juice, followed by a 750-1250 ms intertrial interval. The experiment commenced with a training phase, during which the subjects were trained to perform the task using a set of images that were not employed in the fMRI experiments. In the subsequent test phase, each true-colored object image from the fMRI experiments was paired with the corresponding false-colored object image. In one session, all pairs were presented once, with balanced locations (left or right side), resulting in a total of 120 (15 × 4 × 2) trials per session. Two to four sessions were conducted for each subject each day, depending on the monkey’s performance (M2: 24 sessions, 7 days; M3: 19 sessions, 6 days).

**Data analysis**

We measured the spatial locations of the fixations (≥150 ms) (2) on each completed trial using EyeMMV toolbox (3). We then calculated two parameters to characterize the monkey's preference for true- and false-colored object categories: the proportion of fixation time and proportion of first fixation (4). The proportion of fixation time was calculated by dividing the fixation time in a specific condition by the total fixation time in one trial and then averaged across completed trials within each session. The first fixation proportion was calculated by dividing the number of first fixations in a specific category by the total completed trials within each session.

**Statistical analysis**

We conducted the statistical analyses using Generalized Linear Mixed Models (GLMMs), with Monkey, Session, and Run as random factor.

1. Tomeo OB, Ungerleider LG, Liu N. Preference for Averageness in Faces Does Not Generalize to Non-Human Primates. Front Behav Neurosci. 2017;11:129.

2. Taubert J, Flessert M, Wardle SG, Basile BM, Murphy AP, Murray EA, et al. Amygdala lesions eliminate viewing preferences for faces in rhesus monkeys. Proc Natl Acad Sci U S A. 2018;115(31):8043-8.

3. Krassanakis V, Filippakopoulou V, Nakos B. EyeMMV toolbox: An eye movement post-analysis tool based on a two-step spatial dispersion threshold for fixation identification. Journal of Eye Movement Research. 2014;7(1).

4. Taubert J, Wardle SG, Flessert M, Leopold DA, Ungerleider LG. Face Pareidolia in the Rhesus Monkey. Curr Biol. 2017;27(16):2505-9 e2.
