# Supplementary material for: Object color knowledge representation occurs in the macaque brain despite the absence of a developed language system
Source: PLoS Biol. 2024 Oct 28;22(10):e3002863. doi: 10.1371/journal.pbio.3002863 (PMC11542842; doi:10.1371/journal.pbio.3002863)
Supplement: S2 Text — (DOCX) [file pbio.3002863.s034.docx]

**Color patches defined by the group analysis**

**Definition of color patches**

The same chromatic and achromatic grating contrast as in the single monkey analysis were used to define the color patches at the group level. As shown in S12A Fig, we were able to identify four clusters around V4d, TEO, and TEpd at a threshold of p < 0.01 (uncorrected). Lowering the threshold to p < 0.05 (uncorrected) allowed for the identification of an additional cluster near the boundary between areas TEa and IPa, as indicated by the red solid lines. As mentioned above, the color bias was relatively weak in the right hemisphere. To account for this weakness, we adjusted the luminance contrast of the achromatic grating from 50% to 25% for M1 and M2. This adjustment enabled the identification of two clusters near V4v and TEad at a threshold of p < 0.01, which are marked with white solid lines. However, even with a more lenient threshold (p < 0.05, uncorrected) and adjusted luminance contrast, we were unable to locate color biased clusters near TEav as reported in the previous study (1) and defined by individual level analyses (i.e., TEav_c). This result may not be surprising, considering the inter-animal variability and the relatively small size of this cluster. Then, we defined 4-mm radius spheres centered at the mass of the clusters obtained from the group analysis. For each monkey, the top 50 color-bias voxels within these spheres were selected to yield the final color patches.

**Results**

We conducted the same analyses as the color patches defined on the individual activation maps and found similar results. For example, we found that all the newly defined color patches exhibited a significantly higher response to chromatic gratings compared to achromatic ones (S12B Fig). Additionally, they were able to successfully discriminate among the three chromatic gratings in the MVPA (S12C Fig). Importantly, TEOp_c (a cluster near the posterior region of TEO) was capable of successful classifications among the memory color and true-false color decoding (S12D-F Fig). Unsurprisingly, slight differences were also observed. For example, TEa_c (AFc) defined on the individual monkeys’ activation maps could encode memory color when training on chromatic gratings, but not when defined by the group analysis.

1. Lafer-Sousa R, Conway BR. Parallel, multi-stage processing of colors, faces and shapes in macaque inferior temporal cortex. Nat Neurosci. 2013;16(12):1870-8.
