# Supplementary material for: Object color knowledge representation occurs in the macaque brain despite the absence of a developed language system
Source: PLoS Biol. 2024 Oct 28;22(10):e3002863. doi: 10.1371/journal.pbio.3002863 (PMC11542842; doi:10.1371/journal.pbio.3002863)
Supplement: S3 Text — (DOCX) [file pbio.3002863.s035.docx]

**Color selectivity index**

For each color patch, the color selectivity index was calculated based on responses to chromatic grating and achromatic gratings following Eq 1 (*1*).

$Color selectivity=\frac{Rc-Ra}{Rc+Ra}$ (1)

Where Rc and Ra represent the averaged responses evoked by the unselected chromatic and achromatic gratings when defining color patches, respectively. Note that if either Rc or Ra were negative, a value was added to each of them to ensure that both became zero or positive.
