# Supplementary material for: Object color knowledge representation occurs in the macaque brain despite the absence of a developed language system
Source: PLoS Biol. 2024 Oct 28;22(10):e3002863. doi: 10.1371/journal.pbio.3002863 (PMC11542842; doi:10.1371/journal.pbio.3002863)
Supplement: Attachment — Submitted filename: responses_to_reviews_20240809.docx [file pbio.3002863.s037.docx]

Reviewers’ comments:

Reviewer #2 (Remarks to the Author):

I appreciate the substantial effort the authors have put into revising this paper for the third time. The improvements are noticeable and effective. I have just two brief comments:

1.	While my latest comment on the classification analysis has been fully addressed—thanks to the authors’ diligent work—the second part of the paper still appears weak. Despite the effort to tone down the discussion, it does not, in my view, sufficiently meet the required standards.

>> We appreciate and concur with the reviewer’s insightful comments. In response, we have refined our conclusions regarding the second part of the paper, specifically the section on TP. For example, we have omitted the results related to TP from the Abstract, choosing instead to concentrate on the findings pertaining to color patches.

2.	I concur with the second reviewer regarding the issue of replication across subjects. With only three monkeys, if the results do not convincingly replicate, this may raise concerns about the robustness of the findings.

That said, the paper has significantly improved since its first draft and is worthy of publication in its current form. However, I still harbor reservations about the reliability of the results for a top-tier journal. I understand that this assessment can be subjective, and I would certainly not oppose the publication of this paper in Nature Communications if other reviewers/editors feel differently.

>> Please refer to our responses to Reviewer #3 for clarification regarding the consistency of subjects. Briefly, we achieved significance in three subjects for the major results.

Reviewer #3 (Remarks to the Author):

I do not find the paper suitable for a journal with standards of Nature Comms. As discussed in my earlier reviews, the paper makes a big and important claim, but fails to back it up with evidence. As explained before, replication of the basic finding in all animals is a red line in my standards and I cannot give that standard up.

>> We would like to clarify that in the revised manuscript, we have clearly focused on the results that demonstrate statistical significance at both the group and individual levels.

In the current study, as detailed in the main text, we conducted a series of major analyses focused on color patches and obtained significant results at the group level: 1) classification of grayscale objects with red and green memory colors, 2) memory color decoding by training on chromatic gratings and testing on grayscale objects, and 3) true-false color decoding (Fig. 2). Additionally, we obtained significant results from the two univariate analyses for TP (Fig. 3). Among the abovementioned significant group-level results, we also achieved significance across three subjects in all (only one p-value being marginally significant at 0.0505, see Table R1 below and Fig. 3A-B) but one instance, which was the true-false color decoding analysis. The reviewer’s concern likely arises from this exception. In the true-false color decoding analysis, we achieved significance in only two subjects. However, it is important to note that our major conclusions were not based on this analysis. As discussed in our revised manuscript (Lines 482-486), the limited significance of this analysis may be influenced by the processing of visual perceptual information, which was intentionally matched between the true- and false-colored conditions, particularly within the color patches. Future studies utilizing methods with higher temporal resolution, such as electrophysiological recordings, may provide deeper insights into this aspect.

**Table S1. Consistent results in TEO_c at both the group level and at the individual level in two memory color decoding analyses.**

| Analysis | p-value at the individual level | | |
| --- | --- | --- | --- |
|  | M1 | M2 | M3 |
| Classification of grayscale objects with red and green memory colors | 0.0467 | 0.0107 | 0.0000 |
| Memory color decoding: training on chromatic gratings & testing on grayscale objects | 0.0505 | 0.0000 | 0.0049 |

Notably, in this study, we have presented both group-level and individual-level results to ensure transparency in our findings. To further enhance the robustness of our results, we conducted both ROI analyses and whole-brain analyses. This comprehensive approach allowed us to validate the consistency and reliability of our findings across different analytical methods, thereby strengthening the overall validity of our conclusions.
