# Supplementary material for: Object color knowledge representation occurs in the macaque brain despite the absence of a developed language system
Source: PLoS Biol. 2024 Oct 28;22(10):e3002863. doi: 10.1371/journal.pbio.3002863 (PMC11542842; doi:10.1371/journal.pbio.3002863)
Supplement: Attachment — Submitted filename: responses_to_editors_20240910.docx [file pbio.3002863.s038.docx]

We greatly appreciate the constructive comments from the editors and have closely revised our manuscript accordingly. Below, we provide a point-by-point response to the editors’ comments. The editors’ comments are presented in black, and our responses follow thereafter in red.

Editors’ comments:

1. We would like to suggest a different title to improve readability/accuracy: “Object color knowledge representation occurs in the macaque brain despite the absence of a developed language system”

>> We appreciate the editors’ insightful comments and have accordingly revised our title.

2. Please add the links to the funding agencies in the Financial Disclosure statement in the manuscript details.

>> We acknowledge the editor’s reminder and have added the links to the submission system.

3. Please include the full name of the IACUC/ethics committee that reviewed and approved the animal care and use protocol/permit/project license. Please also include an approval number.

>> We thank the reviewer for this helpful comment. We have included the full name of the IACUC and the approval number in the revised manuscript (Lines 610-612, Page 27) and below.

*“All experimental procedures complied with the US National Institutes of Health Guide for the Care and Use of Laboratory Animals and were approved by the Institutional Animal Care and Use Committee of IBP (IBP-NHP-003).”*

4. DATA POLICY:

You may be aware of the PLOS Data Policy, which requires that all data be made available without restriction: http://journals.plos.org/plosbiology/s/data-availability. For more information, please also see this editorial: http://dx.doi.org/10.1371/journal.pbio.1001797

Note that we do not require all raw data. Rather, we ask that all individual quantitative observations that underlie the data summarized in the figures and results of your paper be made available in one of the following forms:

1) Supplementary files (e.g., excel). Please ensure that all data files are uploaded as ‘Supporting Information’ and are invariably referred to (in the manuscript, figure legends, and the Description field when uploading your files) using the following format verbatim: S1 Data, S2 Data, etc. Multiple panels of a single or even several figures can be included as multiple sheets in one excel file that is saved using exactly the following convention: S1_Data.xlsx (using an underscore).

2) Deposition in a publicly available repository. Please also provide the accession code or a reviewer link so that we may view your data before publication.

Regardless of the method selected, please ensure that you provide the individual numerical values that underlie the summary data displayed in the following figure panels as they are essential for readers to assess your analysis and to reproduce it: 2CEG, 3DEF, S1BC, S2AB, S3ABC, S4ABC, S5AC, S6B, S7ABC, S8ABC, S9AB, S10, S11ABC, S12BCDEF, S13DE, S15BCDEFG, S16AB, S17ABC, S18ABCDEF, S19AB, S20AB, S21ABC, S22Ab, S23, S24ABCD, S25AB and S27ABCD

NOTE: the numerical data provided should include all replicates AND the way in which the plotted mean and errors were derived (it should not present only the mean/average values).

Please also ensure that figure legends in your manuscript include information on where the underlying data can be found, and ensure your supplemental data file/s has a legend.

Please ensure that your Data Statement in the submission system accurately describes where your data can be found.

>> We thank the editor for the thoughtful and patient reminder. We have provided the individual numerical values involved in the figures in the main text and supplementary materials in S1 Data (filename: S1_Data.xlsx). Furthermore, we have made the main experimental data available through public links to provide more information (Open Science Framework: <https://osf.io/e9wju/>). We haven’t made this project public yet. Here’s a private link for your review prior to publication (<https://osf.io/e9wju/?view_only=7a349df4d84c4cb888d71253b7defaa5>).

We have included information on where the underlying data can be found in all figure legends (for main figures: *“The data underlying this figure are available in S1 Data and* [*https://osf.io/e9wju/*](https://osf.io/e9wju/)*.”*; for supplementary figures: *“The data underlying this figure are available in S1 Data.”*). In addition, the S1 Data file also has a legend: *“Data underlying the plots in all figures.”*

We have also updated the Data Statement in the submission system.

5. CODE POLICY

Per journal policy, if you have generated any custom code during the course of this investigation, please make it available without restrictions. Please ensure that the code is sufficiently well documented and reusable, and that your Data Statement in the Editorial Manager submission system accurately describes where your code can be found.

Please note that we cannot accept sole deposition of code in GitHub, as this could be changed after publication. However, you can archive this version of your publicly available GitHub code to Zenodo. Once you do this, it will generate a DOI number, which you will need to provide in the Data Accessibility Statement (you are welcome to also provide the GitHub access information). See the process for doing this here: <https://docs.github.com/en/repositories/archiving-a-github-repository/referencing-and-citing-content>.

>> We thank the editor for the thoughtful and patient reminder. We have made the customized scripts available through public links (Open Science Framework: <https://osf.io/e9wju/>; private link for your review: <https://osf.io/e9wju/?view_only=7a349df4d84c4cb888d71253b7defaa5>). The codes sourced from the toolbox or articles have been appropriately cited in the main text. In accordance with recommendations, we have also deposited both the data and code in Zenodo (<https://zenodo.org/records/13739051>; private link for your review: <https://zenodo.org/records/13739051?preview=1&token=eyJhbGciOiJIUzUxMiJ9.eyJpZCI6IjdkOThmYjVlLWEyYzQtNGNiNC1hYWNiLTc1MzRkMDVmZjc1MyIsImRhdGEiOnt9LCJyYW5kb20iOiJiZGI5YjAwNjk3ZTllYmYzMmVlZDYyMWIxZmQ5NWVkMiJ9.W9lg6Q5M2zmhtPI_Am2TEv5nHrfdIpYy7o-EzjKKW5JhtrZ4QyELq5zismOMwyNkTdRFQE4x06wRiQKLjNr0EQ>). To prevent any confusion, we provide the OSF link in the revision, consistent with other articles published in PLOS Biology. Should Zenodo be deemed more appropriate, we are prepared to update the link accordingly.
